# FOXR1 regulates stress response pathways and is necessary for proper brain development

**DOI:** 10.1371/journal.pgen.1009854

**Published:** 2021-11-01

**Authors:** Andressa Mota, Hannah K. Waxman, Rui Hong, Gavin D. Lagani, Sheng-Yong Niu, Féodora L. Bertherat, Lynne Wolfe, Christine May Malicdan, Thomas C. Markello, David R. Adams, William A. Gahl, Christine S. Cheng, Uwe Beffert, Angela Ho

**Affiliations:** 1 Department of Biology, Boston University, Boston, Massachusetts, United States of America; 2 Bioinformatics Program, Boston University, Boston, Massachusetts, United States of America; 3 NIH Undiagnosed Diseases Program, Common Fund, Office of the Director, National Institutes of Health, and National Human Genome Research Institute, National Institutes of Health, Bethesda, Maryland, United States of America; Columbia University Medical Center, UNITED STATES

## Abstract

The forkhead box (Fox) family of transcription factors are highly conserved and play essential roles in a wide range of cellular and developmental processes. We report an individual with severe neurological symptoms including postnatal microcephaly, progressive brain atrophy and global developmental delay associated with a *de novo* missense variant (M280L) in the *FOXR1* gene. At the protein level, M280L impaired FOXR1 expression and induced a nuclear aggregate phenotype due to protein misfolding and proteolysis. RNAseq and pathway analysis showed that FOXR1 acts as a transcriptional activator and repressor with central roles in heat shock response, chaperone cofactor-dependent protein refolding and cellular response to stress pathways. Indeed, FOXR1 expression is increased in response to cellular stress, a process in which it directly controls *HSPA6*, *HSPA1A* and *DHRS2* transcripts. The M280L mutant compromises FOXR1’s ability to respond to stress, in part due to impaired regulation of downstream target genes that are involved in the stress response pathway. Quantitative PCR of mouse embryo tissues show *Foxr1* expression in the embryonic brain. Using CRISPR/Cas9 gene editing, we found that deletion of mouse *Foxr1* leads to a severe survival deficit while surviving newborn *Foxr1* knockout mice have reduced body weight. Further examination of newborn *Foxr1* knockout brains revealed a decrease in cortical thickness and enlarged ventricles compared to littermate wild-type mice, suggesting that loss of *Foxr1* leads to atypical brain development. Combined, these results suggest FOXR1 plays a role in cellular stress response pathways and is necessary for normal brain development.

## Author summary

Exome sequencing of an individual with severe neurological symptoms including postnatal microcephaly, progressive brain atrophy, and global developmental delay implicated a *de novo* missense variant in the *FOXR1* gene as potentially causative. FOXR1 is a member of the forkhead box (FOX) family of transcription factors with unknown function. Overexpression of FOXR1 in cultured cells show diffuse nuclear localization, while the FOXR1 mutant led to an accumulation of nuclear aggregates due to protein misfolding. As a transcription factor, FOXR1 was found to regulate a large number of genes including those involved in protein folding pathways, while the mutant showed impaired regulation of stress-responsive genes. Although FOXR1 is expressed at low levels in most tissues, we detected *Foxr1* expression in mouse embryonic brain tissue. Using CRISPR gene editing, deletion of the *Foxr1* gene in mice led to reduced survival at birth. Brain pathology of *Foxr1* knockout mice revealed decreased cortical thickness and an enlargement of ventricles. Our data reveal that FOXR1 regulates genes involved in proper protein folding and lack of *Foxr1* in mice is associated with reduced survival and brain pathology consistent with observations found in the human brain.

## Introduction

Neurodevelopmental disorders result from abnormal brain development and the inability to reach cognitive, emotional, and motor developmental milestones. Progress in genomics has advanced the prognosis of human neurodevelopmental disorders and provided insights into the molecular mechanisms of disease [1–3]. While some causal genes are highly penetrant, there are also many rare single-nucleotide changes that have deleterious effects on genes of unknown function. Through exome sequencing, the NIH Undiagnosed Diseases Program (NIH UDP), a clinical site of the NIH Undiagnosed Diseases Network (UDN), identified a variant (M280L) in a single allele of the *FOXR1* gene (forkhead box R1; NM_181721.2) in an individual with severe neurological symptoms including postnatal microcephaly, progressive brain atrophy, and global developmental delay.

FOXR1 is a member of the evolutionarily conserved forkhead box (Fox) family of transcription factors named after the ectopic head structures observed in mutants of the *Drosophila* gene *forkhead* (*fkh*) [4–6]. Mutations in the *Drosophila fkh* gene cause defects in head fold involution during embryogenesis, resulting in a characteristic spiked head appearance in adult flies. Since the discovery of *fkh*, hundreds of *Fox* genes have been identified in organisms ranging from yeasts to humans, making it one of the largest but least explored families of higher eukaryotic transcription factors (reviewed in [7–8]). All members of the *Fox* gene family of transcription factors are monomeric, helix-turn-helix proteins that harbor a core fkh DNA-binding domain comprised of three α-helices connected *via* a small β-sheet to a pair of loops resembling butterfly wings or a “winged-helix” [9–11]. Despite the high degree of conservation identity in the DNA-binding domain, Fox proteins bind different target sequences with great specificity. Fox proteins affect transcriptional regulation of large array of genes directing major developmental processes such as cell proliferation and cell fate specification [9,12–14]. Human genetic analyses show several *FOX* genes have important biological functions associated with brain development; these include *FOXG1* (potential determinant of forebrain size; [15–17]) and *FOXP2* (vocal learning; [18–20]). Further, mutations in *FOXG1*, *FOXC2*, *FOXL2*, *FOXP1* and *FOXP2* have profound effects on human brain development including microcephaly, intellectual impairments, and language disorders [21–25].

FOXR1, also known as FOXN5 (forkhead box N5) or DLNB13, is a 292 amino acid protein that contains a fkh DNA-binding domain [26]. The human *FOXR1* and rat *Foxr1* gene consist of six exons with conserved exon-intron structure, indicating that FOXR1 is well-conserved between human and rat genomes [27]. The Genome-based tissue expression consortium indicate that *FOXR1* is expressed in the human brain and reproductive organs [28]. The Human Brain Transcriptome shows that *FOXR1* is expressed in all brain regions during embryonic and postnatal development and its expression level in the brain is maintained throughout life (https://hbatlas.org). Furthermore, *in situ* hybridization showed that mouse *Foxr1* expression was present in all brain regions and enhanced within cellular nuclei, consistent with the human tissue expression profile based on the Allen Brain Atlas [29]. However, little is known about the function of FOXR1. Several studies have shown that mouse *Foxr1* is involved in spermiogenesis [30]. In addition, several point mutations within human *FOXR1* have been shown to be associated with a variety of carcinomas, although functional characterization of these oncogenic *FOXR1* mutants has not been performed [31–33]. Recently, *Foxr1* was found to be an essential maternal–effect gene in zebrafish that is required for proper cell division and survival [34].

Here, we report a human neurodevelopmental disorder associated with a rare variant in *FOXR1*. We demonstrate that the *de novo* missense M280L variant decreases FOXR1 protein expression and exhibits nuclear puncta aggregates in HEK293T cells, suggesting that impaired FOXR1 function can be pathogenic. In addition, we show that the FOXR1 M280L mutant has a compromised ability to respond to stress, in part due to impaired regulation of downstream target genes that are involved in the stress response pathway. Further, our analysis revealed *Foxr1* knockout mice exhibit a severe survival deficit. Surviving newborn *Foxr1* knockout mice show cortical thinning and enlarged ventricles suggesting that the architecture of the mammalian brain is dependent on *Foxr1*.

## Results

### Exome sequencing identified an individual with developmental delay carrying a *de novo* missense variant in *FOXR1*

**Fig 1:**
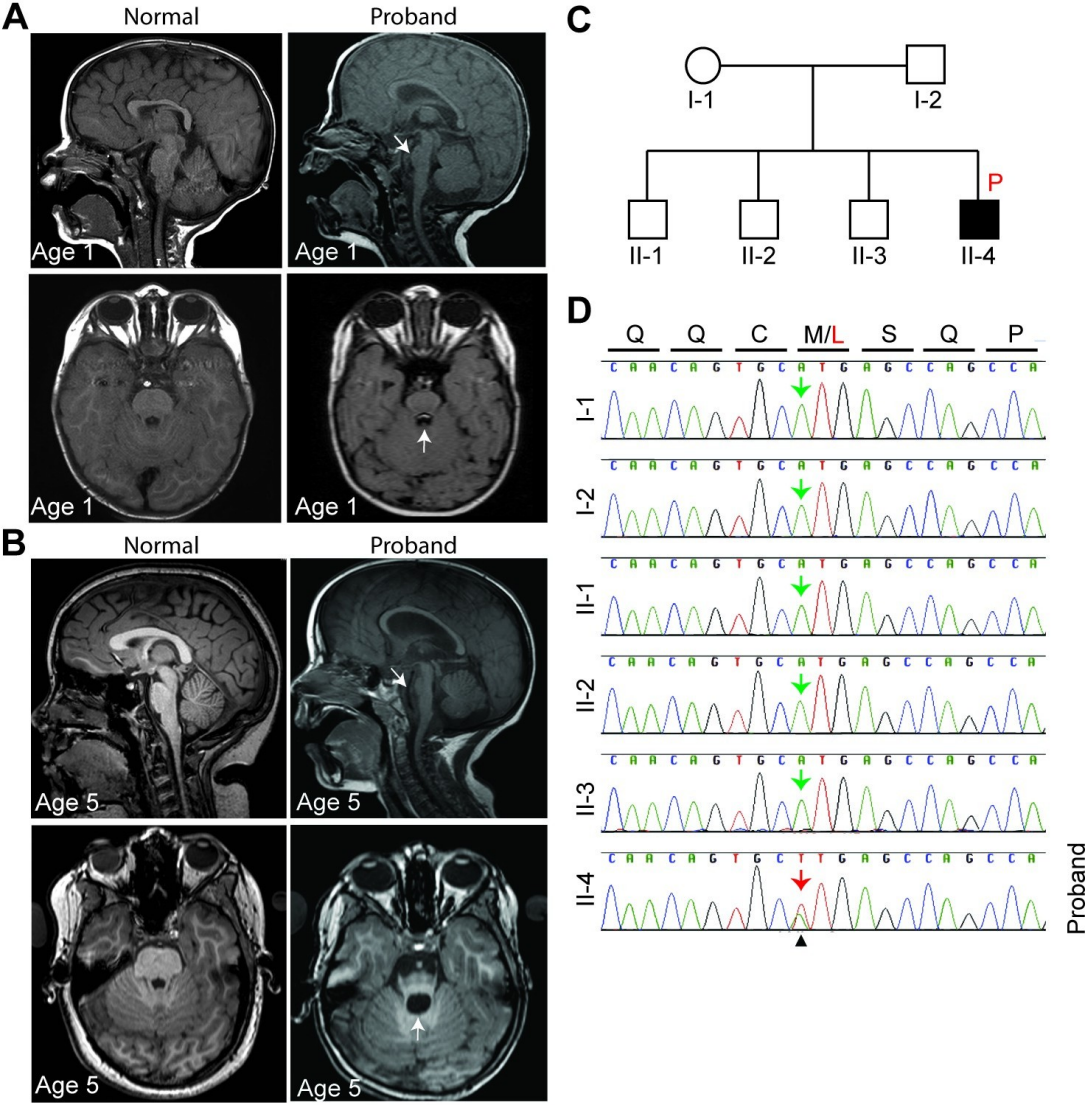
*De novo FOXR1* missense variant in a proband with microcephaly and brain atrophy. **(A)** MRI scans of mid-sagittal (top) and horizontal (bottom) view of normal age-matched and the proband at 1 year old. **(B)** MRI scans of mid-sagittal (top) and horizontal (bottom) view of normal age-matched and the proband at 5 years old. Arrow on mid-sagittal images indicate hypoplasia of the pons in the proband. Also, arrow on horizontal view show dilation of ventricle in the proband compared to age-matched normal individual. **(C)** Pedigree of the family where the letter P in red (black square) indicates the proband. **(D)** Sanger sequence analysis confirming the *de novo FOXR1* variant. Sequence chromatograms demonstrate the presence of the heterozygous variant in the proband, II-4 (indicated by the red arrow) and the reference allele in both parents and siblings (green arrows). Letters on top indicate amino acid residues (Q = glutamine, C = cysteine, M = methionine, L = leucine, S = serine, P = proline).

The NIH UDP identified a proband with severe neurological symptoms including postnatal microcephaly, progressive brain atrophy, and severe muscle hypotonia from early infancy. Brain MRI showed progressive hypoplasia in the cerebral cortex, pons and cerebellum and ventricular enlargement from age 1 to 5 compared to age-matched normal MRI brain scans (Fig 1A and 1B). The proband also exhibits growth delay, decreased body weight, short stature, scoliosis, hip dysplasia, ankle clonus, and bell-shaped thorax (S1 Table). Ophthalmic abnormalities include optic atrophy, cortical visual impairment, and retinitis pigmentosa. Neuromuscular abnormalities include hyperactive deep tendon reflexes, joint hypermobility, severe muscle hypotonia, and poor head control. In addition, the proband has myopathic facies, preauricular pits, anteverted nares and low set ears.

Exome sequencing was performed on the proband and the siblings and parents who are all unaffected. Three likely pathogenic candidate genes, rapamycin and FKBP12 target (*RAFT1*), ATPase Na^+^/K^+^ transporting subunit alpha 3 (*ATP1A3*), and *FOXR1* were identified. RAFT1 functions as a kinase that regulates cell growth, proliferation, motility, and survival [35–36]. The proband has a homozygous *RAFT1* missense variant, but the EXAC database identified an unaffected individual with the same *RAFT1* variant. The second candidate, *ATP1A3*, maintains plasma membrane sodium and potassium gradients [37]. Investigations discovered an individual with the same variant who displays a mild phenotype involving learning disability and episodes of dizziness. Variants in ATP1A3 were considered to have contributed to the final phenotype and were returned to the family as a partial diagnosis (OMIM disorders 182350 and / or 128235). The last candidate is a *de novo* missense variant in *FOXR1*, a gene of unknown function, and the variant was not identified in the siblings or parents (Fig 1C). The heterozygous *de novo* nonsynonymous variant results in a methionine-to-leucine substitution at position 280 (M280L) and was confirmed by Sanger sequencing (Fig 1D). M280 is found in the C-terminal segment of the FOXR1 protein, which is downstream of the DNA-binding domain. M280 is highly evolutionarily conserved, from mammals, birds, reptiles to frogs and zebrafish (S1 Fig). In addition, the M280L variant is predicted to be damaging and disease-causing based on scores of Combined Annotation Dependent Depletion (score of 29.9 where a score of 30 means that the variant is in the top 0.1% of deleterious variants in the human genome), PolyPhen-2 (score: 0.994/1.0), and Mutation Taster (score: 0.99/1.0). Although a preliminary diagnosis implicating the *ATP1A3* variant for this patient has been made, a synergistic contribution from additional variants including the FOXR1 M280L variant cannot be ruled out.

### The FOXR1 M280L mutant leads to a decrease in FOXR1 protein expression

**Fig 2:**
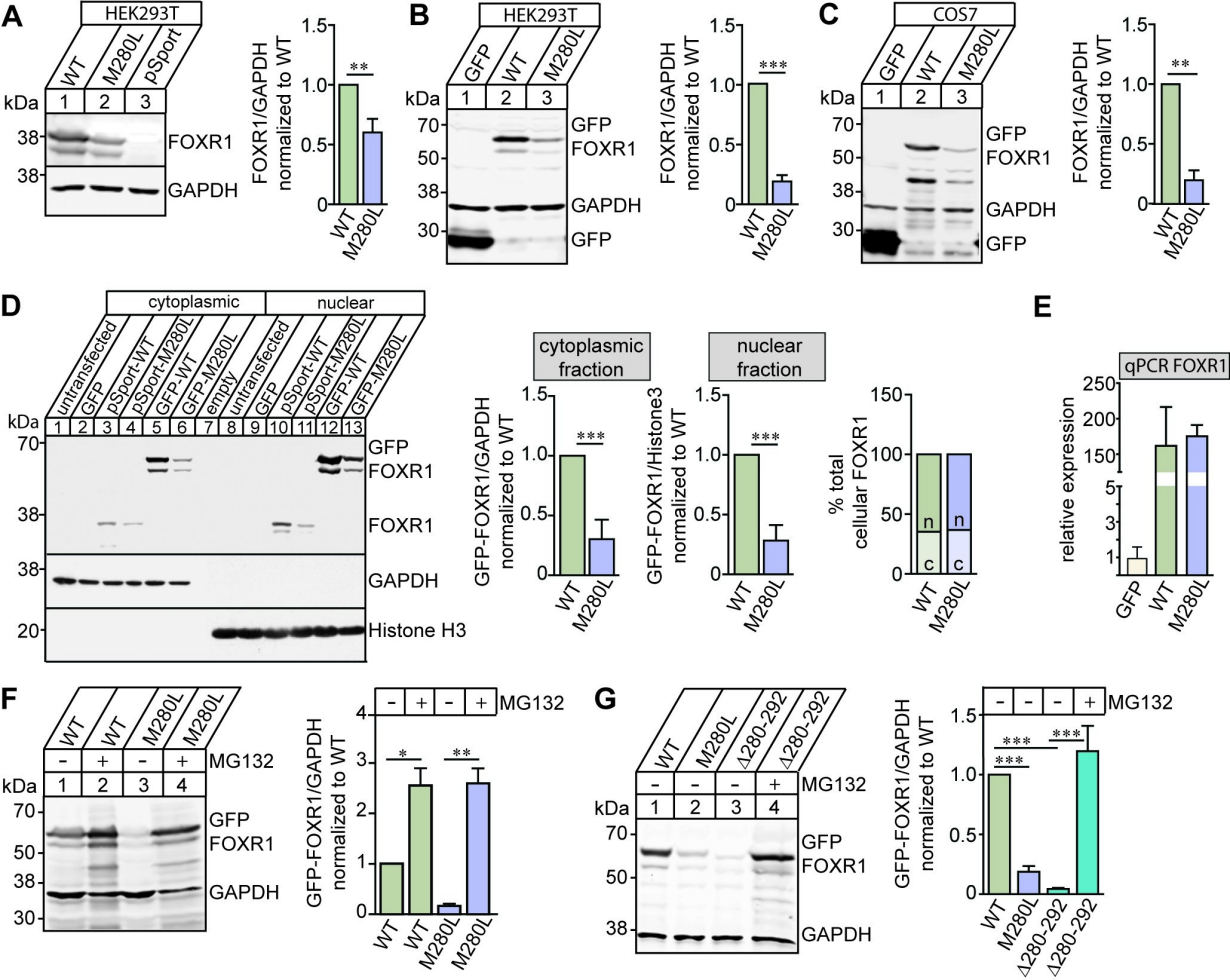
The M280L mutant destabilizes FOXR1 protein. **(A)** Representative immunoblots and quantitative analysis of FOXR1 from HEK293T cells transfected with pCMV-SPORT6 human FOXR1 WT or M280L mutant. GAPDH served as a loading control. Graph represents FOXR1 over GAPDH normalized to WT. Unpaired *t*-test (n = 4 independent experiments, ** p = 0.0025). **(B)** Representative immunoblot and quantitative analysis of FOXR1 from HEK293T cells transfected with GFP, GFP-tagged human FOXR1 WT or M280L mutant. GAPDH served as a loading control. Graph represents FOXR1 over GAPDH normalized to WT. Unpaired *t*-test (n = 5 independent experiments, *** p < 0.0001). **(C)** Representative immunoblot and quantitative analysis of FOXR1 from COS7 cells transfected with GFP, GFP-tagged human FOXR1 WT or M280L mutant. GAPDH served as a loading control. Graph represents FOXR1 over GAPDH normalized to WT. Unpaired *t*-test (n = 4 independent experiments, ** p = 0.0013). **(D)** Representative immunoblots and quantitative analysis of cytoplasmic (c) and nuclear (n) fractions of FOXR1 from HEK293T cells transfected with pCMV-SPORT6 or GFP-tagged human FOXR1 WT or M280L. GAPDH and Histone H3 served as cytoplasmic and nuclear loading markers, respectively. Graph represents FOXR1 over GAPDH normalized to WT. Unpaired *t*-test (n = 5 independent experiments, *** p < 0.0001). The percentages of total cellular FOXR1 in the cytoplasmic and nuclear fractions were determined. **(E)** Quantitative PCR (qPCR) to quantify *FOXR1* mRNA levels from HEK293T cells transfected with GFP, GFP-tagged human FOXR1 WT or M280L mutant. Graph represents relative *FOXR1* mRNA expression normalized to GFP. One-way ANOVA Tukey’s multiple comparisons test (n = 3 independent experiments). **(F)** Representative immunoblot and quantitative analysis of FOXR1 from HEK293T cells transfected with GFP-tagged human FOXR1 WT or M280L mutant. Protein stability was monitored by quantitative immunoblotting after blocking with proteasome inhibitor MG132. Graph represents FOXR1 over GAPDH normalized to untreated WT. One-way ANOVA Tukey’s multiple comparisons test (n = 3 independent experiments, * p = 0.0245, ** p = 0.0003). **(G)** Representative immunoblot and quantitative analysis of FOXR1 from HEK293T cells transfected with GFP-tagged human FOXR1 WT, M280L mutant or FOXR1 C-terminal truncation mutant lacking the last 12 amino acids (Δ280–292). Protein stability was monitored for FOXR1 Δ280–292 mutant by blocking proteasome degradation with MG132. GAPDH served as a loading control. Graph represents FOXR1 over GAPDH normalized to untreated WT. One-way ANOVA Tukey’s multiple comparisons test (n = 3 independent experiments, *** p < 0.0001).

To examine whether the FOXR1 M280L mutant was properly expressed *in vitro*, we transiently transfected FOXR1 wild-type (WT) or the M280L mutant in HEK293T or COS7 cells and immunoblotted for FOXR1 or GFP-tagged FOXR1 protein. FOXR1 levels were significantly decreased in the M280L mutant (Fig 2A, 2B and 2C). Since *FOXR1* is a transcription factor, we next tested whether the M280L mutant affects FOXR1 nuclear localization in HEK293T cells transfected with either untagged or GFP-tagged FOXR1 WT or M280L. Western blot analysis demonstrated that both FOXR1 WT and M280L protein are localized in both cytoplasmic and nuclear fractions with higher levels found in the nuclear fraction (Fig 2D). However, protein levels of the M280L mutant was reduced compared to FOXR1 WT in both cytoplasmic and nuclear fractions.

We next investigated whether the decrease in FOXR1 levels in the M280L mutant was due to transcription or protein stability changes. In HEK293T-transfected cells, we detected equal amounts of *FOXR1* mRNA levels of FOXR1 WT and M280L, indicating that decreased M280L protein levels are not due to decreased transcription (Fig 2E). To measure protein stability, we blocked the proteasome pathway by treating transfected HEK293T cells with MG132, a cell-permeable proteasome inhibitor. Protein levels of both FOXR1 WT and M280L were approximately the same after proteasome inhibition. This suggests that the M280L variant destabilizes the FOXR1 protein, likely due to protein misfolding which, make it susceptible to proteolysis and degradation through the proteasome pathway (Fig 2F).

Finally, we investigated whether the short C-terminal tail containing M280 is necessary for protein stabilization. We generated a FOXR1 C-terminal truncation mutant lacking the last 12 amino acids from M280 (Δ280–292). Indeed, HEK293T cells transfected with GFP-tagged Δ280–292 have decreased FOXR1 protein levels, which increased following MG132 treatment, suggesting that the FOXR1 C-terminal tail is critical for FOXR1 protein stability (Fig 2G).

### FOXR1 M280L induces a nuclear aggregate phenotype

**Fig 3:**
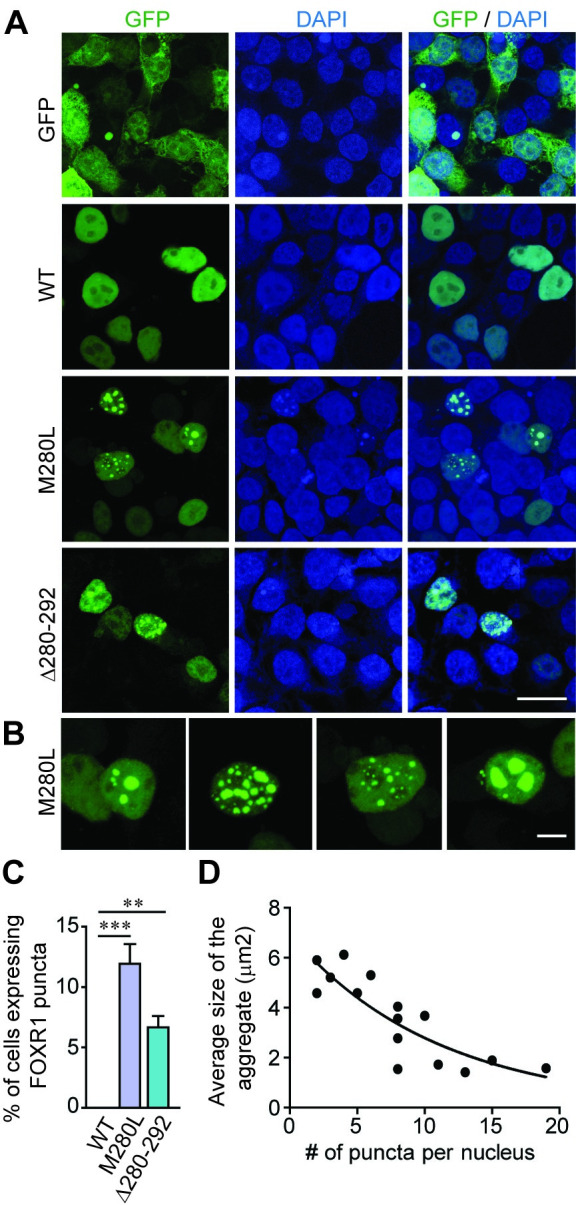
The M280L variant induces nuclear puncta phenotype. **(A)** Fluorescence images of HEK293T cells transfected with GFP or GFP-tagged human FOXR1 WT, M280L or Δ280–292 mutants. DAPI (blue) served as a nuclear marker. Scale bar = 20 μm. **(B)** Fluorescence images of HEK293T cells transfected with GFP-tagged M280L mutant showing a range of nuclear puncta phenotypes. Scale bar = 5 μm. **(C)** Quantitative analysis of the percentage of cells showing FOXR1 puncta phenotype. One-way ANOVA Tukey’s multiple comparisons test (n = 3 independent experiments, ** p = 0.0048, *** p = 0.0002). **(D)** Correlation analysis of the average size of the aggregate to the number of puncta per nucleus.

To examine whether the M280L mutant alters the cellular localization of FOXR1, we transfected HEK293T cells with GFP, GFP-tagged FOXR1 WT, M280L, or Δ280–292 mutant. Immunostaining for GFP shows FOXR1 WT mainly in a diffuse pattern in the nucleus, co-localizing with DAPI, a nuclear marker (Fig 3A). In contrast, about 13% of cells transfected with the M280L mutant form discrete nuclear puncta (Fig 3B and 3C). We observed a similar phenotype in COS7 cells transfected with the M280L variant (S2 Fig). In nuclei containing >15 puncta, the average size of individual puncta was <2 μm^2^, whereas nuclei containing <5 puncta had aggregates of >4 μm^2^ (Fig 3D). These results suggest that the larger puncta may form by coalescing from small nuclear foci. In addition, cells transfected with the FOXR1 Δ280–292 mutant displayed a similar nuclear puncta pattern, suggesting that the C-terminal tail of FOXR1 is necessary for proper folding of the protein.

### Identification of novel FOXR1-dependent transcripts by RNA sequencing analysis

**Fig 4:**
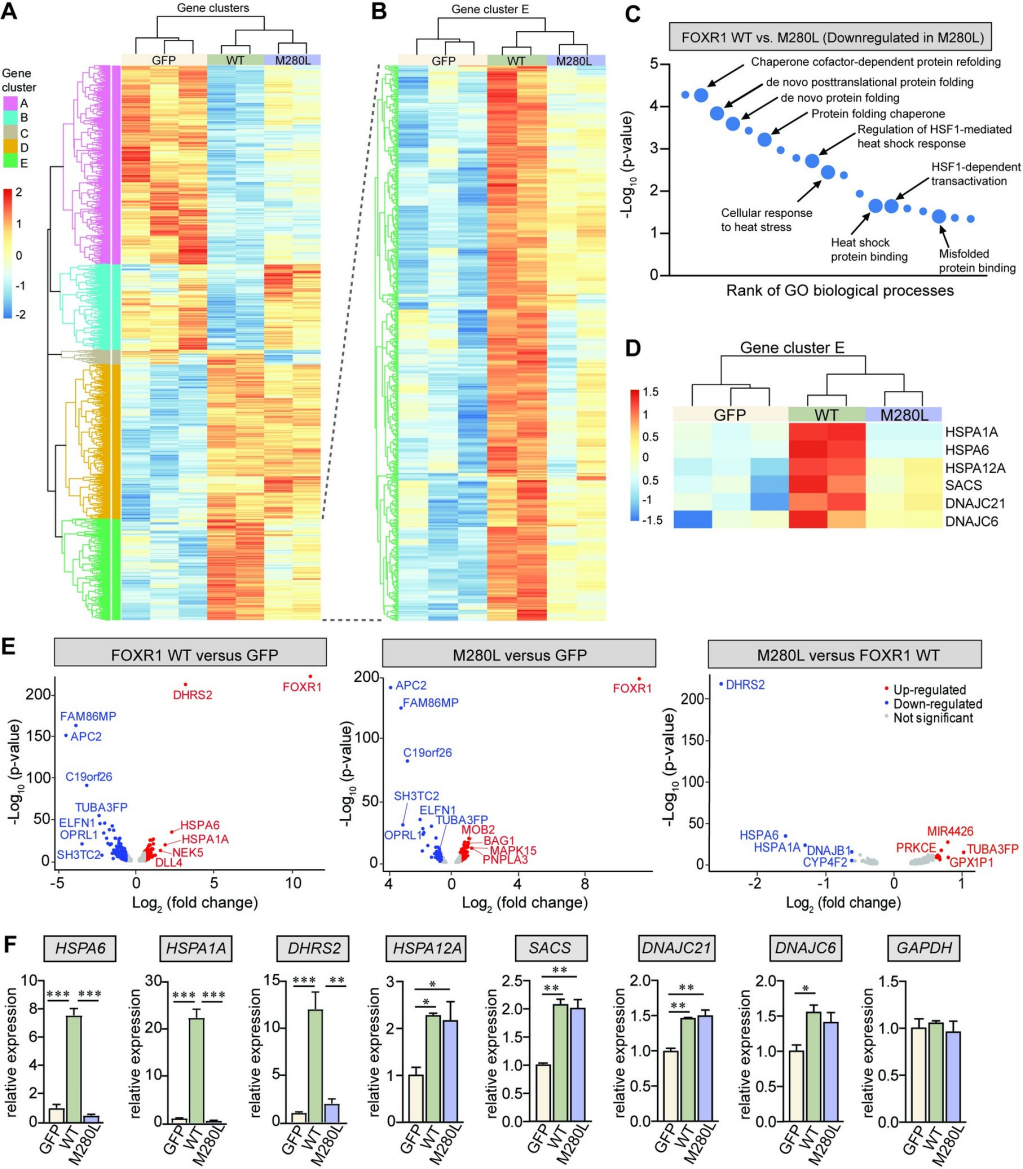
RNAseq analysis of FOXR1 wild-type and M280L mutant. **(A)** Heatmap of hierarchical clustering indicates differentially-expressed genes (rows) between GFP, GFP-tagged FOXR1 WT and M280L (fold-change > 2, p < 0.05). Red indicates up-regulated genes and blue indicates down-regulated genes. **(B)** Heatmap of gene cluster ‘E’ indicates differentially-expressed genes (rows) that are upregulated in FOXR1 WT and down-regulated in M280L compared to WT. **(C)** Distribution of gene ontology (GO) terms annotated in biological processes of highly-regulated genes in FOXR1 WT and down-regulated in M280L. **(D)** Heatmap of gene cluster ‘E’ highlighting several chaperone proteins that were differentially expressed in FOXR1 WT and down-regulated in M280L. **(E)** Volcano plots of differentially expressed genes between FOXR1 WT versus GFP control, M280L versus GFP and FOXR1 WT versus M280L. Significantly up-regulated genes are in red while down-regulated genes are in blue. Non-significant genes are in gray. **(F)** Quantitative real-time PCR verifying the RNAseq analysis showing FOXR1 drives expression of *HSPA6*, *HSPA1A* and *DHRS2* and are misregulated in the M280L mutant. Graph represents relative expression. One-way ANOVA Tukey’s multiple comparisons test (n = 3 independent experiments, * p < 0.05, ** p < 0.005, *** p < 0.0001).

To identify target genes regulated by FOXR1 and to investigate the effect of FOXR1 M280L, we performed an unbiased transcriptomic screen by RNA sequencing (RNAseq) in HEK293T cells transiently transfected with GFP, GFP-tagged FOXR1 WT or M280L. Principal component analysis showed that the three groups clustered separately excluding experimental covariates and batch effects (S3A Fig). We plotted a heat map of the log (-2) fold change for all the differentially-expressed genes (DEGs) and delineated five coherent clusters (Fig 4A). Differential gene expression analysis between GFP and FOXR1 WT transfected cells identified 2644 DEGs of which 1315 (49.7%) were upregulated and 1329 (50.3%) were downregulated transcripts (Figs 4A and S3B). To determine the effect of FOXR1 M280L, we compared WT and M280L, and identified 735 DEGs of which 561 (76.3%) were upregulated and 174 (23.7%) were downregulated (S3B Fig). We paid special attention to those transcripts whose levels showed a 2-fold increase in FOXR1 WT and a decrease in M280L as delineated in cluster E (Fig 4B).

Gene ontology (GO) analysis for biological processes within cluster E shows genes involved in the heat shock response. This cluster contains genes that are functionally-related to negative regulation of inclusion body assembly, chaperone cofactor-dependent protein refolding, *de novo* protein folding, cellular response to stress, and regulation of HSF1-mediated heat shock response where these are enriched in FOXR1 WT and downregulated in M280L (Figs 4C and S4). Based on the volcano plots that summarize both the expression fold-change and the statistical significance, the upregulated genes in response to FOXR1 WT and downregulated in M280L include *HSPA1A* and *HSPA6* (both members of the Hsp70 family of heat shock proteins, Hsps), and *DHRS2* (Dehydrogenase/Reductase SDR Family Member 2, a mitochondrial reductase enzyme) (Fig 4D and 4E). These proteins play roles in protecting against oxidative stress. In addition, when we examined the volcano plot between M280L relative to GFP, we found overlapping transcripts between M280L and GFP and between WT and GFP (Fig 4E). In fact, this was confirmed by a high Pearson’s correlation (*r* = 0.96) examining the log_2_ (fold change) between WT with GFP and M280L with GFP suggesting the M280L mutation functions as a hypomorphic loss of function mutation due to reduced levels of the FOXR1 protein (S3C Fig).

Quantitative real-time-PCR (qRT-PCR) supported the RNAseq data for *HSPA6*, *HSAPA1A* and *DHRS2* (Fig 4F), confirming upregulation of gene expression in FOXR1 WT but not in the M280L mutant. Other Hsps such as *SACS*, *DNAJC21* and *DNAJC6* were increased in both FOXR1 WT and M280L groups. Not all members of the Hsp70 family were misregulated in the M280L mutant; for example, the *HSPA12A* transcript was found to be upregulated in both FOXR1 WT and the M280L mutant. These results indicate that FOXR1 drives expression of specific Hsps and an important NADPH-dependent reductase enzyme that is likely related to cytoprotective pathways alleviating oxidative stress. To determine whether the DEGs contain consensus sequences for FOXR1 response elements [14], we examined the promoter regions of DEGs for each cluster except for cluster C which comprised of only a few genes. Each cluster contains a subset of DEGs carrying the FOXR1 consensus element which may be direct targets of FOXR1, supporting FOXR1 playing a role as both a transcriptional activator and repressor (S3D Fig).

### FOXR1 controls gene expression of heat shock chaperones and an antioxidant NADPH-dependent reductase

**Fig 5:**
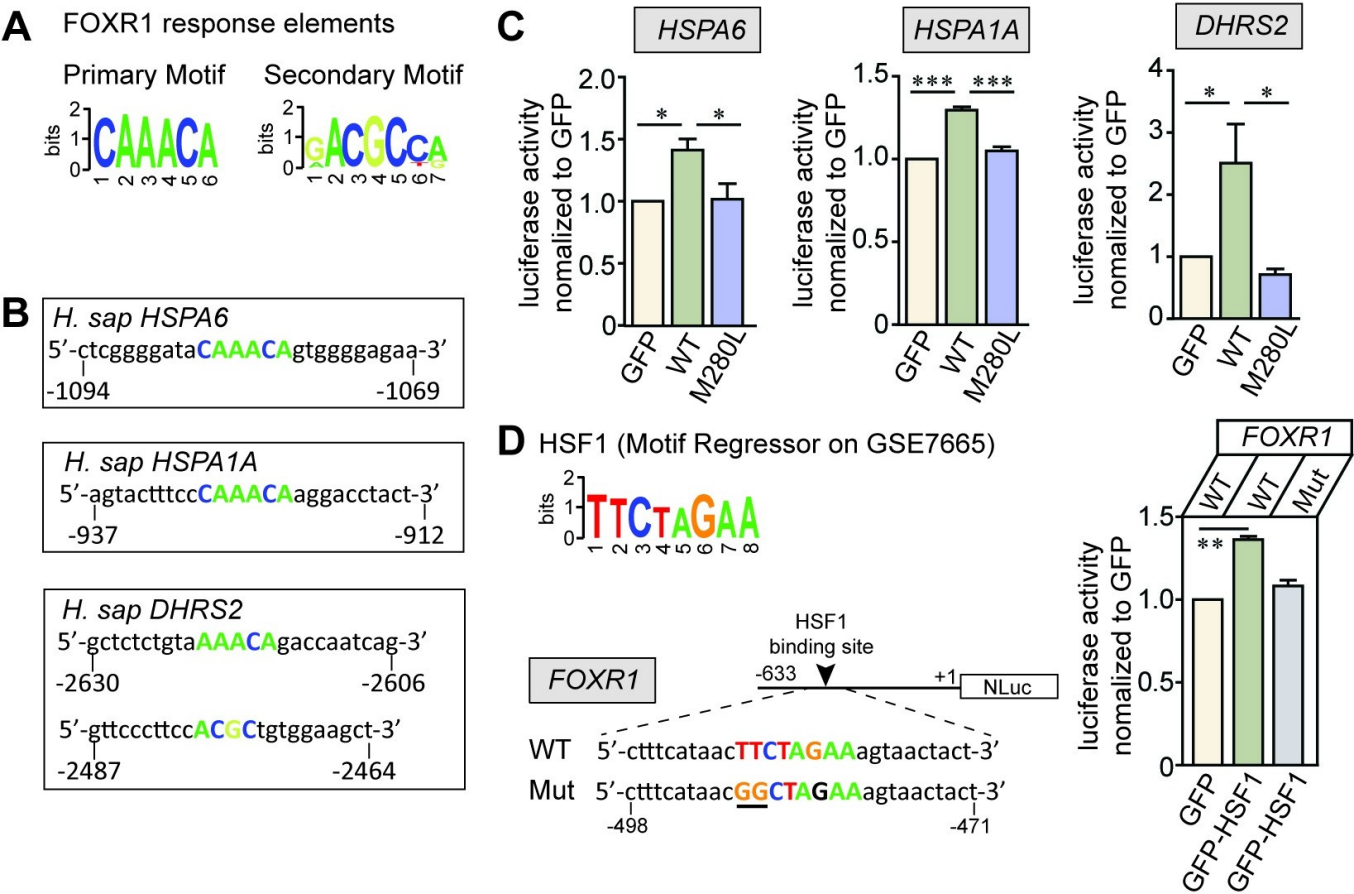
Human DNA binding-site motifs bound by FOXR1. **(A)** FOXR1 response elements showing consensus primary and secondary sequences bound by FOXR1 (adapted from [14]). **(B)** Putative FOXR1 response elements are denoted in the promoters of three of the top-regulated FOXR1-targeted genes: *HSPA6*, *HSPA1A* and *DHRS2*. **(C)** Dual luciferase reporter assays where GFP control, FOXR1 WT or M280L were co-transfected into HEK293T cells with the corresponding *HSPA6*, *HSPA1A* and *DHRS2* luciferase reporters. Data are plotted as luciferase activity normalized to GFP control. One-way ANOVA Tukey’s multiple comparisons test (n = 3 independent experiments, * p < 0.05, *** p < 0.0002). **(D)** Consensus primary sequences bound by HSF1. The putative HSF1 response elements are denoted in the promoter of *FOXR1*. Dual luciferase reporter assays where GFP control or GFP-HSF1 were co-transfected in HEK293T cells with corresponding *FOXR1* WT or Mut luciferase reporter. *FOXR1* mutant (Mut) consists of the HSF1 response elements in FOXR1 where the two TT residues in FOXR1 WT are mutated to GG (underlined). Data was plotted as luciferase activity normalized to GFP control. One-way ANOVA Tukey’s multiple comparisons test (n = 3 independent experiments, ** p = 0.0062).

To determine whether *HSPA6*, *HSPA1A* and *DHRS2* are directly regulated by FOXR1, we manually performed a *de novo* motif analysis of target promoters to identify consensus DNA-binding sites upstream of the ATG start site (Figs 5A and S5). We found strong consensus sequences for FOXR1 response elements [14] within the promoter regions of at least three of the top FOXR1-regulated genes, *HSPA6*, *HSPA1A* and *DHRS2* (Fig 5B). To determine whether FOXR1 regulates the expression of these three genes through interaction with their promoter sequences, we utilized a dual luciferase system under the control of proximal upstream regions of human *HSPA6* (-1119 to -113 bp), *HSPA1A* (-1053 to -210 bp) or *DHRS2* (-3329 to -2313 bp) and co-transfected with either GFP control, FOXR1 WT or M280L mutant in HEK293T cells. We found that *HSPA6*, *HSPA1A*, and *DHRS2* are activated by FOXR1 WT but not by M280L, indicating that these promoter regions contain FOXR1 responsive sequences and are targets of FOXR1 WT (Fig 5C).

Expression of many Hsps is known to be regulated by the transcription factor heat shock factor 1 (HSF1), which has a high affinity for *cis*-acting DNA sequence elements, including the heat shock elements (HSEs) found in the promoters of HSF-responsive genes such as Hsp70 proteins [reviewed in 38]. There is also precedence that HSF1 target genes extend beyond molecular chaperones. For example, in *C*. *elegans*, the protective effects of reduced insulin signaling requires both HSF1 and the FOXO transcription factor, *DAF-16*, to prevent damage by protein misfolding and to promote longevity [39–41]. Based on the GO analysis for biological processes, transcripts that were upregulated in FOXR1-transfected cells were genes related to regulation of HSF1-mediated heat shock response (S4 Fig). We therefore, tested whether HSF1 may regulate FOXR1 since we identified a consensus sequence for HSF1 binding within the promoter region of FOXR1 (Fig 5D). Utilizing a dual luciferase system under the control of an upstream region of human FOXR1 (-633 to +1 bp), FOXR1 was found to be activated by GFP-HSF1 (Fig 5D). However, HSF1-mediated FOXR1 activation was not observed when the HSF response element in FOXR1 was mutated from ***TT***CTAGAA to ***GG***CTAGAA (Mut) *in vitro*, indicating that human FOXR1 is a target of HSF1, which may be regulated by cellular stress.

### FOXR1 expression is increased in response to cellular stress

**Fig 6:**
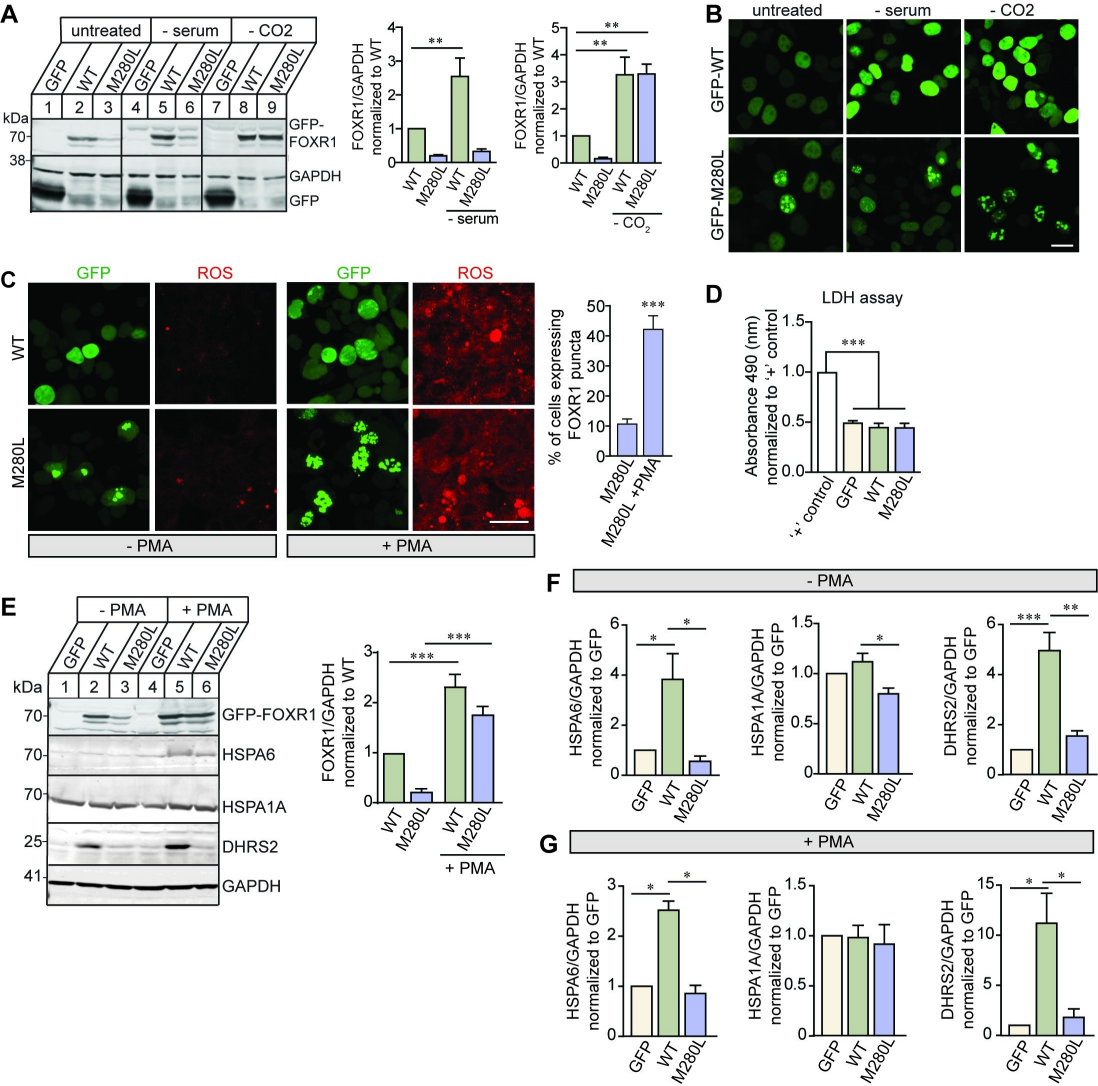
FOXR1 expression is increased in response to cellular stress. **(A)** Representative immunoblots and quantitative analysis for FOXR1 from HEK293T cells transfected with GFP, GFP-tagged FOXR1 WT or M280L mutant in response to serum and CO_2_ deprivation. GAPDH served as loading control. Graph represents FOXR1 over GAPDH normalized to untreated WT. One-way ANOVA Tukey’s multiple comparisons test (n = 4 independent experiments, ** p < 0.0051). **(B)** Fluorescence images of HEK293T cells transfected with GFP-tagged human FOXR1 WT or M280L in response to serum and CO_2_ deprivation. Scale bar = 20 μm. **(C)** Fluorescence images of HEK293T cells transfected with GFP-tagged human FOXR1 WT or M280L and treated with PMA, a NADPH oxidase activator known to enhance reactive oxygen species (ROS). Cells were fixed after 24 hours of treatment and assessed for ROS generation using CellROX, a photostable ROS sensor. Scale bar = 20 μm. Quantitative analysis of the percentage of cells expressing FOXR1 puncta phenotype. Unpaired *t*-test (n = 4 independent experiments, *** p < 0.0001). **(D)** Lactate dehydrogenase (LDH) levels from conditioned media of HEK293T cells following PMA treatment. Positive control is a set of cells treated with the lysis buffer. Data are expressed based on the absorbance reading at 490 nm normalized to positive control. One-way ANOVA Tukey’s multiple comparisons test (n = 3 independent experiments, *** p < 0.0001). **(E)** Representative immunoblots and quantitative analysis of HEK293T cells following PMA treatment showing an increase in FOXR1 expression. Graph represents FOXR1 over GAPDH normalized to untreated WT. One-way ANOVA Tukey’s multiple comparisons (n = 5 independent experiments, *** p < 0.0001). **(F)** Quantitative analysis of HSPA6, HSPA1A and DHRS2 protein levels from HEK293T cells transfected with GFP, GFP-tagged human FOXR1 WT or M280L. Graph represents protein of interest over GAPDH normalized to GFP. One-way ANOVA Tukey’s multiple comparisons (n = 3–5 independent experiments, * p < 0.05, ** p < 0.005, *** p < 0.0005). **(G)** Quantitative analysis of HSPA6, HSPA1A and DHRS2 protein levels from HEK293T cells transfected with GFP, GFP-tagged human FOXR1 WT or M280L and treated with PMA. Graph represents protein of interest over GAPDH normalized to GFP. One-way ANOVA Tukey’s multiple comparisons (n = 2–3 independent experiments, * p < 0.05).

Because FOXR1 regulates expression of *HSPA6* and *HSPA1A* transcripts and they are also direct targets of HSF1, we hypothesized that FOXR1 expression might be directly regulated following stress-induced paradigms. We induced cellular stress using two different paradigms: serum deprivation (metabolic stress for 24 hours) and CO_2_-deprivation (oxidative stress for 24 hours). Cells transfected with FOXR1 WT exhibited a 2.5- and 3.3-fold increase in FOXR1 protein levels under serum- and CO_2_-deprivation, respectively, when compared to the non-stressed condition (Fig 6A). The increase in FOXR1 protein levels coincided with an increase in nuclear FOXR1 (Fig 6B). In contrast, FOXR1 M280L protein levels also exhibited a 3.3-fold increase under CO_2_-deprivation but not during serum-deprivation, indicating that the M280L mutant may be sensitive to different types of environmental stressors. In fact, the number of nuclear aggregates in cells transfected with the M280L mutant in response to CO_2_-deprivation was increased but not in response to serum-deprivation (S1 Video).

To further explore the relationship between FOXR1 and oxidative stress, we treated FOXR1-transfected HEK293T cells with phorbol 12-myristate 13-acetate (PMA), a pharmacologic NADPH oxidase activator known to enhance reactive oxygen species (ROS) through a protein kinase C-mediated pathway [42]. We assessed ROS generation by fluorescence imaging using CellROX, a photostable ROS sensor. Consistent with other stress paradigms, PMA enhanced ROS generation in HEK293T cells transfected with FOXR1 WT and M280L (Fig 6C). PMA enhanced the diffuse FOXR1 fluorescence in the nucleus of HEK293T cells transfected with FOXR1 WT. The number of nuclear aggregates in cells transfected with the M280L mutant was increased by 3.9-fold compared to non-PMA treatment (Fig 6C and S2 Video), suggesting ROS-induced aggregation of mutant FOXR1 protein in response to stress. To determine whether ROS-induced aggregation of FOXR1 protein is cytotoxic, we measured the amount of lactate dehydrogenase (LDH) released into the medium. While the PMA induced some ROS toxicity, we found no LDH changes between cells transfected with GFP alone and GFP-tagged FOXR1 WT or between FOXR1 WT and M280L, indicating that the nuclear aggregates were not cytotoxic (Fig 6D).

We found FOXR1 protein levels were increased 2.3- and 1.8-fold in cells transfected with FOXR1 WT and M280L mutant after PMA treatment, respectively (Fig 6E). Concomitantly, we found an increase in both HSPA6 and DHRS2 protein levels in cells transfected with FOXR1 WT (Fig 6F and 6G). HSPA6 levels were increased in response to PMA treatment in the M280L mutant. In contrast, we did not observe any changes in DHRS2 protein expression levels in cells transfected with M280L regardless of PMA treatment. In addition, while we observed a significant increase in *HSPA1A* mRNA levels in cells transfected with FOXR1 WT (Fig 4), we did not detect any changes in HSPA1A protein levels in cells transfected with FOXR1 WT or M280L. However, we did consistently see a decrease in HSPA1A protein levels in cells transfected with M280L compared to FOXR1 WT, but this difference disappeared when cells were treated with PMA.

### FOXR1 nuclear puncta in M280L mutant are insoluble

**Fig 7:**
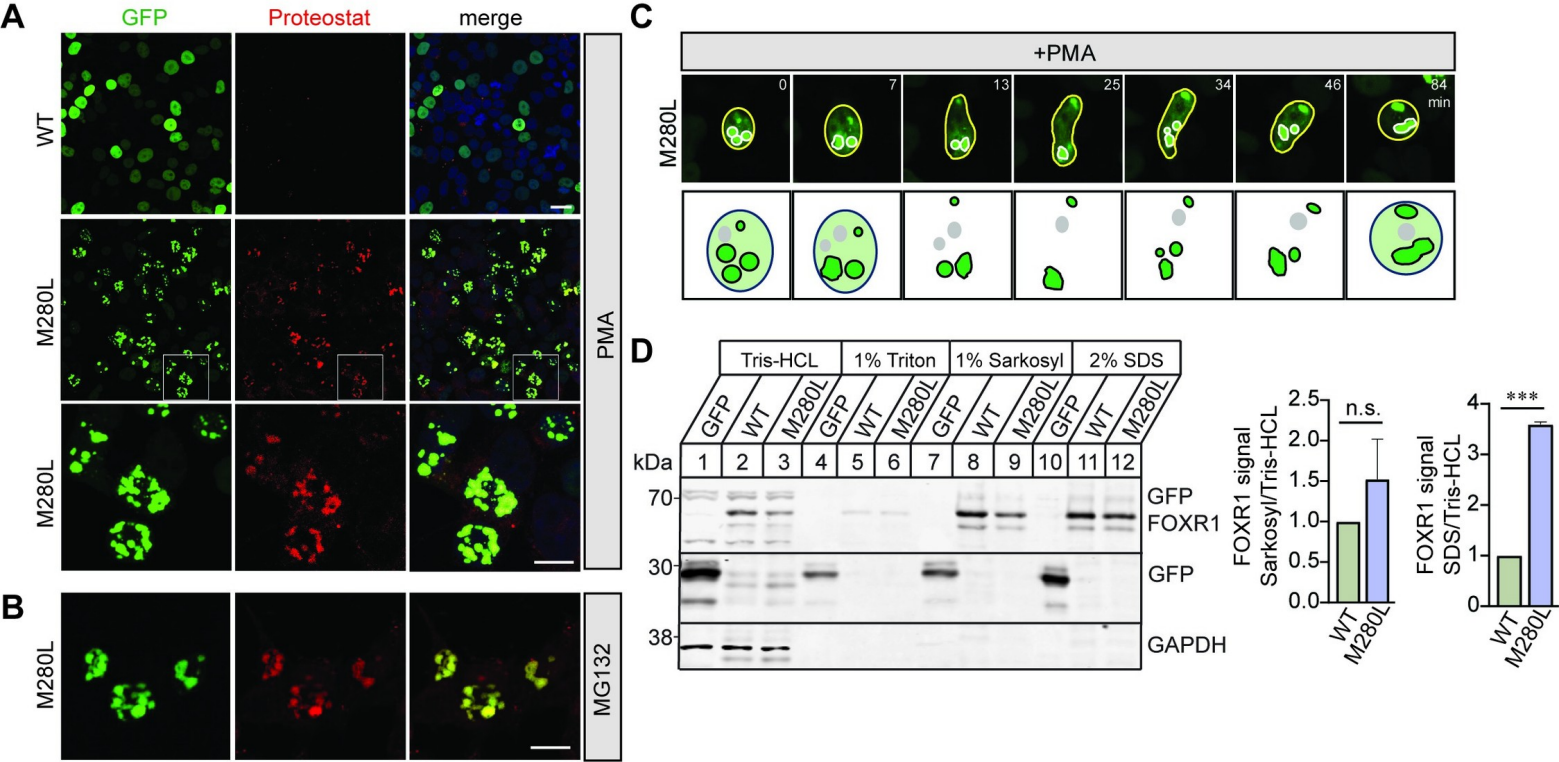
M280L nuclear aggregates are insoluble misfolded proteins. **(A)** Fluorescence images of HEK293T cells transfected with GFP-tagged human FOXR1 WT or M280L and treated with PMA. Cells were fixed after 24 hours of treatment and immunolabeled with Proteostat marker. White square box in the middle panels indicate images presented in the bottom panel at higher magnification. Top and middle panels, scale bar = 20 μm. Bottom panels, scale bar = 10 μm. **(B)** Fluorescence images of HEK293T cells transfected with GFP-tagged M280L and treated with MG132. Cells were fixed after 24 hours of treatment and immunolabeled with Proteostat marker. Scale bar = 10 μm. **(C)** Time-lapse imaging of HEK293T cells transfected with GFP-tagged M280L. Top panel represents images showing nuclear aggregates undergoing extensive movements and fusions. Bottom panel illustrates schematic drawings of the fusion events. Scale bar = 5 μm. **(D)** FOXR1 was sequentially extracted with Tris-HCl, Triton X-100, Sarkosyl and SDS. Quantification shows that the amount of FOXR1 in the sarkosyl fraction was not significant (n.s.) between WT and M280L. However, the SDS fraction was significantly higher in the M280L mutant when compared to the overall Tris-HCl total fraction. One-way ANOVA Tukey’s multiple comparisons (n = 2 independent experiments, *** p = 0.0003).

To determine whether the nuclear puncta that form in HEK293T cells transfected with the M280L mutant were aggresomes, which are known to serve as storage bins for misfolded or aggregated proteins [43], transfected HEK293T cells were treated with PMA and stained with the Proteostat dye. The dye detects misfolded and aggregated proteins in cells. We found bright punctate staining for proteostat-positive aggregates colocalized with the nuclear puncta in cells expressing the M280L mutant but not in FOXR1 WT (Fig 7A). These results were similar in transfected cells expressing M280L that were treated with the cell-permeable proteasome inhibitor MG132, further supporting that the M280L variant destabilizes FOXR1 protein and forms nuclear aggregates (Fig 7B).

Misfolded proteins often expose their hydrophobic domains, leading to aggregation [44–45]. In addition, most aggregated proteins tend to coalesce and form large deposits such as aggresomes or inclusion bodies [46–47]. Previous studies have shown that nuclear and cytoplasmic aggregates of poly-Q proteins such as ataxin-1 are dynamic and exchange their components whereas ataxin-3 are immobile [48–49]. In fact, time-lapse live cell imaging of HEK293T cells transfected with GFP-tagged M280L showed that the nuclear aggregates are quite dynamic and undergo extensive movements and fusions, with small aggregates moving toward each other and fusing to form larger aggregates (Fig 7C and S3 Video).

Another criterion of misfolded proteins deposited within aggresomes is that they are largely detergent insoluble [46,50–53]. Thus, we examined the biochemical properties of M280L aggregates versus FOXR1 WT, testing protein lysates from HEK293T cells transfected with GFP, GFP-tagged FOXR1 WT or M280L for their solubility in different detergents. Protein extracts were sequentially extracted by Tris-HCl buffer, Tris-HCl buffer containing 1% Triton-X100, 1% Sarkosyl, and finally by 2% SDS. The amount of FOXR1 extracted in each fraction was assessed by immunoblotting for GFP-FOXR1. GFP-FOXR1 WT was detected in Tris-HCl soluble, Sarkosyl soluble, and SDS soluble fractions but was not present in the Triton X-100 fraction, suggesting that the majority of the FOXR1 WT protein was soluble and, not associated with membrane-bound proteins (Fig 7D). However, the majority of M280L was detected in the SDS fraction and not in the Sarkosyl fraction indicating a significant portion of the protein was insoluble and aggregating, which is consistent with the increased aggregation shown by the Proteostat immunolabeling.

### *Foxr1* knockout mice exhibit cortical thinning and ventricular enlargement

**Fig 8:**
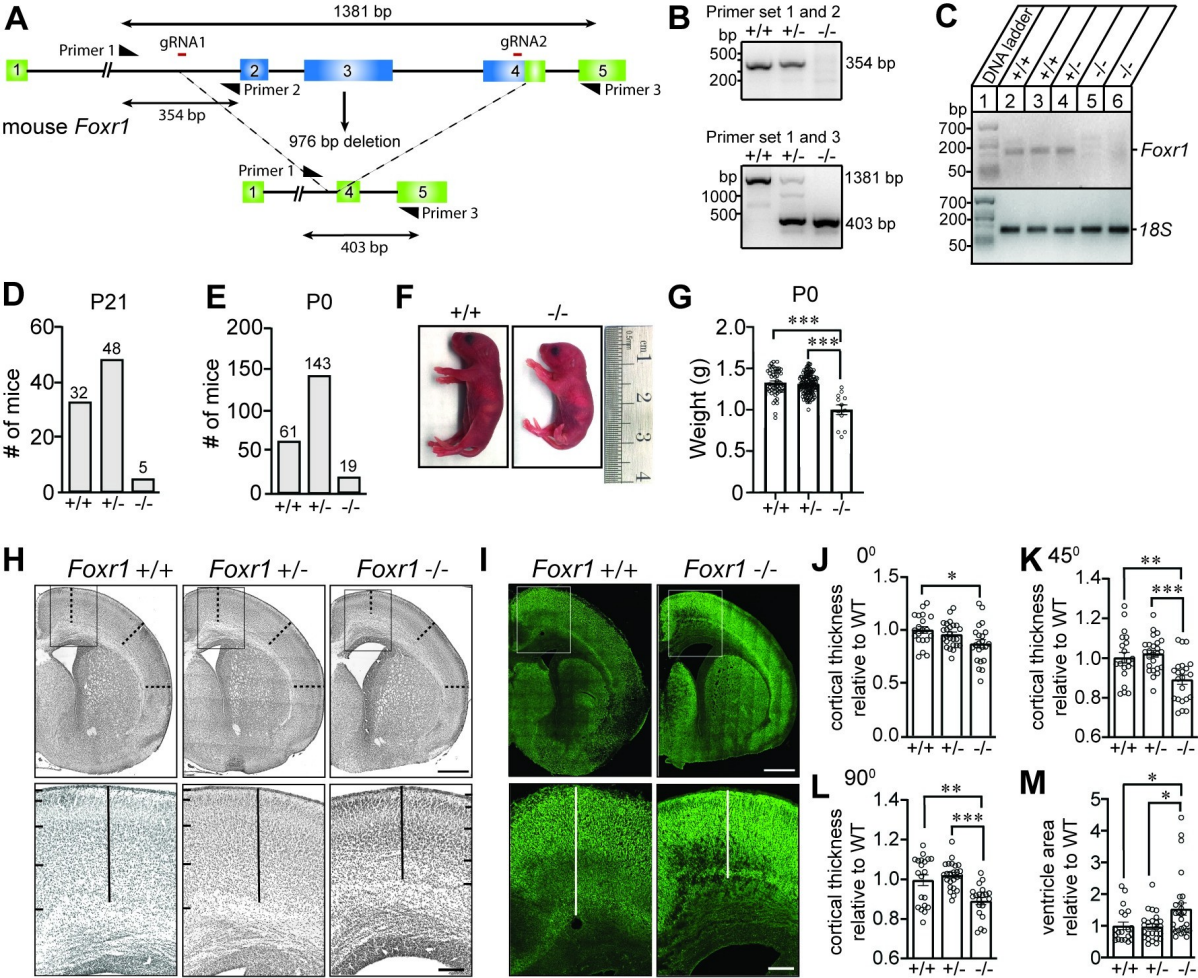
*Foxr1* knockout mice exhibit cortical thinning and enlarged ventricles. (A) Representative schematic view of the CRISPR/Cas9 targeting strategy used for generating *Foxr1* knockout mice. The *Foxr1* locus consists of 5 exons (colored boxes), and introns (black lines). Two gRNAs (red lines), one upstream located in intron 1 and one downstream located within exon 4 were used to target the *Foxr1* gene and remove 976 nucleotides encompassing exons 2, 3 and part of exon 4 (blue box). Primers are represented by black half arrowheads to indicate the relative locations of forward (primer 1) and reverse genotyping primers (primers 2 and 3). **(B)** Representative PCR genotyping result for primer set 1 and 2 (indicated in panel A) to detect *Foxr1* wild-type allele (354 bp). Below is a representative PCR genotyping result for primer set 1 and 3 to detect *Foxr1* wild-type (1381 bp), heterozygous (1381 for wild-type and 403 bp for knockout), and knockout alleles (403 bp). **(C)** RT-PCR of *Foxr1* (175 bp) and 18S ribosomal transcripts (129 bp) from brains of wild-type and *Foxr1* knockout mice. **(D)** Genotype analysis of number of offspring obtained from *Foxr1* heterozygous crossings at postnatal day 21 (P21). **(E)** Genotype analysis of number of newborn offspring obtained from *Foxr1* heterozygous crossings at postnatal day 0 (P0). **(F)** Lateral view of wild-type and *Foxr1* knockout neonates showing a decrease in size in the *Foxr1* knockout mutant. **(G)** Body weight measurements (in grams) for *Foxr1* wild-type (n = 53), heterozygous (n = 120) and knockout mice (n = 12) at postnatal day 0 showing a decrease in *Foxr1* knockout mice. One-way ANOVA Tukey’s multiple comparisons (*** p < 0.001). **(H)** Nissl stain brain sections of *Foxr1* wild-type (+/+), heterozygous (+/-) and knockout (-/-) mice. Top panel shows coronal sections anterior to bregma. Dashed lines represent cortical measurements at 0°, 45° and 90° (relative to the midline) to pia surface. White box indicates higher magnification for the bottom panels. Scale bar = 500 μm. Bottom panels represents higher magnification of the cortex where the vertical line indicates cortical thickness measurements. Side bars demarcates the different cortical layers. Scale bar = 125 μm. **(I)** MAP2 immunostaining of brain sections of *Foxr1* wild-type and knockout mice. Top panel shows coronal sections anterior to bregma. White box indicates higher magnification for the bottom panels. Scale bar = 500 μm. Bottom panels represents higher magnification of the cortex where the vertical line indicates cortical thickness measurements. Scale bar = 125 μm. **(J-L)** Quantification of cortical thickness from pooled brain sections of 4 wild-type, 4 heterozygous and 4 *Foxr1* knockout mice at 0°, 45° and 90° (relative to the midline) to pia surface, respectively. Graph represents relative thickness normalized to wild-type (WT). One-way ANOVA Tukey’s multiple comparisons (0°, * p = 0.0165; 45°, ** p = 0.0033, *** p = 0.0003; 90°, ** p = 0.0019, *** p <0.0001) **(M)** Quantification of ventricle area from pooled brain sections of 4 wild-type and 4 *Foxr1* knockout mice. Graph represents relative thickness normalized to wild-type (WT). One-way ANOVA Tukey’s multiple comparisons * p = 0.04 wild-type and knockout; p = 0.02 heterozygous and knockout.

Human FOXR1 shares 66% amino acid sequence identity with its mouse homologue (S6A Fig). Using primers specific for mouse *Foxr1*, we demonstrated by qPCR that *Foxr1* mRNA was detected in several tissues, including heart, liver, lung, and higher expression in the brain at embryonic day 17 (S6B Fig). To better understand the role of FOXR1 in mammalian brain development, we investigated *Foxr1* function in the mouse brain by analyzing mice that lack the *Foxr1* gene using the CRISPR/Cas9 gene editing system. Single guide RNAs (gRNAs) were designed to target intron one and the majority of exon 4, thus destroying the exon 4 splice acceptor (Fig 8A). This leads to a deletion of exons 2, 3 and majority of exon 4 resulting in a 976 bp deletion from Chr9:44435486 to 44436461. Homozygous *Foxr1* knockout mice were generated from mating heterozygous *Foxr1* mutant mice. Genomic tail DNA was isolated and genotyping was performed using PCR and validated by Sanger sequencing with primer pairs amplifying within the first intron to annotate *Foxr1* wild-type allele and primer pairs amplifying from the first intron to exon 5 to annotate the nucleotide position spanning the entire *Foxr1* deletion region (Figs 8A and 8B and S6C). Analysis of *Foxr1* transcript level by RT-PCR confirmed loss of *Foxr1* expression in the brains of *Foxr1* knockout mice compared to wild-type and heterozygous littermate controls (Fig 8C).

Mating between *Foxr1* heterozygous mice deviated from the normal Mendelian expected 1:2:1 ratio of *Foxr1*^+/+^: *Foxr1*^+/-^: *Foxr1*^-/-^ at postnatal day 21 (χ2 = 18.576, df = 2, p < 0.001). Of the 85 mice, 32 were *Foxr1*^+/+^, 48 were *Foxr1*^+/-^ and only 5 were *Foxr1*^-/-^, thus displaying a severe survival deficit with only ~23.5% of *Foxr1* knockout mice surviving to postnatal day 21 (Fig 8D). To determine whether deletion of *Foxr1* could be lethal either during embryonic development or within the first postnatal weeks, we analyzed the genotypes of offspring from *Foxr1* heterozygous crossings immediately after birth at postnatal day 0 (P0). Of the 223 P0 mice, 61 were *Foxr1*^+/+^, 143 were *Foxr1*^+/-^ and 19 were *Foxr1*^-/-^ (χ2 = 33.619, df = 2, p < 0.001). We observed ~34% of *Foxr1* knockout mice surviving at P0, indicating that a majority of the *Foxr1* knockout mice perish during embryonic development (Fig 8E). In addition, surviving newborn *Foxr1* knockout mice look smaller and weigh 24.5% less compared to their littermates (Fig 8F and 8G).

We next investigated the effect of *Foxr1* deletion by analyzing the brains of newborn *Foxr1* mice by performing serial sectioning and stained for Nissl and MAP2 immunolabeling (Fig 8H and 8I). We analyzed serial coronal sections of *Foxr1* wild-type, heterozygous and knockout brains at different anatomical locations (anterior to bregma, bregma, and posterior to bregma) taking cortical measurements at three different angles (0°, 45°, and 90° from midline) to the pial surface (S7 Fig). Comparison of pooled serial brain sections revealed a ~11.3% thinner cortical plate in *Foxr1* knockout mice compared to wild-type littermates (Fig 8J, 8K and 8L). In addition, we found ventricles are enlarged by 34.6% in *Foxr1* knockout mice compared to wild-type littermates (Figs 8M and S7). *Foxr1* heterozygous mice did not exhibit any histological abnormalities including cortical thinning or ventricular enlargement.

## Discussion

The UDN has identified an individual presenting with severe neurological symptoms and linked a missense variant in the *FOXR1* gene as a potential variant underlying the genetic etiology of the rare neurodevelopmental disorder (Fig 1). Certainly, the presence of likely-disease causing variants in the second gene, *ATP1A3* complicate the ascertainment of the phenotypic contributions of FOXR1. However, the overall severity of the clinical course is uncharacteristic of *ATP1A3*-related disease reported to date. For this reason, we hypothesize that FOXR1, based on the work we describe, is an additional contributor to the final phenotype. The single *de novo* missense variant in *FOXR1* converts a highly conserved methionine residue at amino acid 280 to leucine and was predicted to be damaging and disease-causing based on several web-based applications. Indeed, we found the M280L variant in FOXR1 leads to a robust decrease in FOXR1 protein levels that is due to protein instability (Fig 2). Protein levels of both FOXR1 WT and M280L were approximately the same after proteasome inhibition, suggesting that the M280L mutant destabilizes FOXR1 protein, likely due to protein misfolding making it susceptible to proteolysis and degradation through the proteasome pathway. In support of this finding, we found the M280L variant formed discrete nuclear puncta that colocalize with the Proteostat dye, which recognizes misfolded and aggregated proteins compared to the diffuse nuclear pattern localization in FOXR1 WT (Figs 3 and 7). The M280L nuclear puncta displayed characteristic features of most aggregated proteins wherein smaller foci coalesce to form larger aggregates that are detergent-insoluble, thus impairing the function of FOXR1. We found the C-terminal sequence of FOXR1 is important for determining protein stability. A FOXR1 C-terminal truncation mutant lacking the last 12 amino acids from M280 (Δ280–292) mimics the M280L phenotype, thus suggesting that the M280L variant most likely affects protein structure or protein-protein interactions critical for protein stability.

Here we identified target genes regulated by FOXR1 based on an unbiased transcriptomic screen using RNAseq in HEK293T cells (Fig 4). DEGs which contain a FOXR1 consensus sequence in their promoters suggest that FOXR1 acts as a transcriptional activator and repressor. The most highly upregulated genes in response to FOXR1 WT, and downregulated in M280L, include two members of the Hsp70 family (*HSPA1A*, *HSPA6)* and a mitochondrial reductase enzyme, *DHRS2*. Each of these proteins play a role in mediating the protective cellular response that relieve oxidative stress. In addition, the top FOXR1-regulated genes *HSPA6*, *HSPA1A* and *DHRS2* contain FOXR1 response elements within their promoter regions. Luciferase assays suggest *HSPA6*, *HSPA1A* and *DHRS2* are targets of FOXR1, and M280L abolishes its ability to activate the expression of these target genes. However, additional chromatin immunoprecipitation data will be necessary to show direct binding to the promoters of these target genes. Also, we found that the increased expression of DEGs, *HSPA6*, *HSPA1A* and *DHRS2* was much higher in the RNAseq experiments compared to the luciferase assays. This could stem from the fact that we only cloned a subset of promoter sequence close to the transcription start site for the luciferase assay. As such, there may be other important regulatory elements further upstream that are necessary for full activation of these genes, including proteins that need to bind to other co-regulatory genomic sequences.

Cells respond to environmental stressors though the activation of specific physiological pathways that increase the abundance or activity of chaperone proteins which prevent protein misfolding to protect the proteome and maintain proteostasis [54–57]. One important mechanism is the induction of Hsp expression, such as the large Hsp70 family of proteins which help maintain proteostasis by acting as molecular chaperones during periods of acute cellular stress [58–60]. It is well-established that HSF regulates the expression of several Hsps during times of stress where HSF binds to heat shock elements within the promoter regions of Hsps [61]. However, there is now growing evidence that the Fox family of transcription factors also influences Hsp expression. For example, the FOXO subfamily of transcription factors plays an important role in protecting organisms against stress [62–64]. Both *FOXO* genes in *Drosophila* (*dFOXO*) and in *C*. *elegans* (*DAF16*) are transcriptional activators for *Hsp70* and small *Hsp* genes, respectively that contribute to maintaining proteostasis in response to oxidative stress. DAF16 maintains proteostasis in *C*. *elegans* by transcriptionally increasing a subset of small *Hsp* genes important in DAF-16 dependent lifespan extension [39,65]. *Drosophila* dFOXO also induces transcription of Hsp genes in response to oxidative stress, conferring resistance to ROS [66]. Mammalian FOXO3 and FOXM1 orchestrate programs of gene expression that regulate oxidative stress resistance by upregulating catalase and MnSOD, enzymes involved in the detoxification of reactive oxygen species [63,64,67,68]. Here, we show that FOXR1 protein levels is increased in response to metabolic and oxidative stress that also increase HSPA6 and DHRS2 protein levels. We demonstrated that HSF1 binds to the FOXR1 promoter and induces its transcription, suggesting that FOXR1 is a target of HSF1. Perhaps HSF is a master transcription factor responding to stress and cross-talk with FOXR1 serves to fine tune transcription of target genes in response to specific stress stimuli.

Human genetic analyses show that several FOX transcription factors have important biological functions in brain development and mutations in *FOX* genes have profound effects on development and function of the brain. FOXG1, formerly named Brain Factor-1 (BF-1), is one of the earliest transcription factors expressed in nervous cell types and tissues. FOXG1 is primarily expressed in the telencephalon and *Foxg1* knockout mice showed severe microcephaly with a reduction in size of the cerebral hemispheres [69]. Mechanistically, FOXG1 interacts with the global transcriptional corepressors of the Groucho/transducing-like Enhancer of the split (TLE) family suggesting that FOXG1 acts as a transcriptional repressor coordinating the control of neural progenitor cell proliferation with the timing of differentiation [70]. Disruption of *FOXG1* in humans leads to brain abnormalities including microcephaly and agenesis of the corpus callosum [17,24,71]. In addition, human mutations in both FOXP1 and FOXP2 lead to severe speech and cognitive impairments [18,19,25,72–74] and, both genes have also been linked to autism spectrum disorders [74–77]. To directly address the role of *FOXR1* in brain development, we examined mice with a null mutation in *Foxr1* using CRISPR/Cas9 gene editing. We found a majority of homozygous null *Foxr1* mutants die perinatally. In addition, *Foxr1* knockout mice display cortical thinning and ventricular enlargement compared to littermate wild-type and heterozygous controls, thus suggesting that *Foxr1* is necessary for survival and normal brain development (Fig 8). Since the proband is heterozygous for the M280L mutation, the proband may resemble the heterozygous *Foxr1* mice in some respects. We did not find any histological abnormalities in *Foxr1* heterozygous mice. Based on the qPCR data, *Foxr1* expression in heterozygous mice was similar to that of wild-type mice. This suggests that instability of the M280L mutation likely leads to lower functional protein levels than in a heterozygous individual. In addition, we cannot exclude the possibility that the M280L mutation could be a dominant-negative phenotype wherein some fraction of the protein within the nuclear aggregates is functional.

FOXR1 is not endogenously expressed in HEK293T or COS7 cells. As such, a limitation of this study is that it is based on ectopic overexpression models to examine FOXR1 function. However, the RNAseq analysis in HEK293T cells transfected with FOXR1 WT may provide insight for future studies. For example, some of the upregulated genes were involved in ribosome biogenesis such as the ribosome biogenesis regulator 1 (*RRS1*) and nervous system development (*MTURN*, *PDZD8*, *PTPRZ1*, *NOTCH2*). Since HEK293T cells originate from neural crest cells, this might explain the expression of several neuron-specific genes. Ribosome biogenesis is a key driver in neurodevelopment and dysregulated ribosomal biogenesis results in neurodevelopmental syndromes that present with microcephaly, autism, intellectual deficits, and/or progressive neurodegeneration [78]. Also, ribosome assembly is an energy-demanding process, and alteration of any steps in ribosomal biogenesis leave cells highly prone to proteotoxic stress that triggers rapid activation of a specific stress pathway that coordinately upregulates heat shock target genes [79]. It is possible that FOXR1 plays a role in protection against proteotoxic stress during ribosome assembly which is essential during brain development. We posit that FOXR1 is a transcription factor that regulates critical genes necessary during brain development which are involved in balancing growth and protein homeostasis. Therefore, understanding how FOXR1 regulates the transcription of genes and how this influences brain development are important questions to address in future experiments.

## Materials and methods

### Ethics statement

Research ethics approval for this study was provided by the National Human Genome Research Institute (NHGRI), Institutional Review Board (#76-HG-0238). All described study participants signed consents for the NHGRI, Institutional Review Board approved protocol #76-HG-0238. For participants under 18 years of age, consent was obtained from the parents of the participant.

### Proband enrollment and consent

The proband was evaluated at the National Institutes of Health Undiagnosed Diseases Program (NIH UDP) and was enrolled in the protocol, approved by the National Human Genome Research Institute Institutional Review Board. The parents of the proband provided written informed consent for medical and genetic studies designed to reach medical diagnoses. The MRI images of the Proband and age-matched normal de-identified images originate from other UDP cases.

### Exome sequencing

Exome sequencing was performed using genomic DNA extracted from peripheral whole blood samples from the study participant and family members after informed consent onto an institutional review board approved protocol (76-HG-0238). Exome capture was carried out using manufacturer protocols using the TruSeq Exome Enrichment Kit (Illumina, San Diego, CA) and sequenced on the HiSeq 2000 Sequencing System (Illumina). Alignment to the human genome reference sequence (UCSC assembly hg19, NCBI build 37) was carried out using the Efficient Local Alignment of Nucleotide Data algorithm (*Eland*, Illumina, Inc) as described previously [80]. Briefly, paired-end (PE) reads were aligned independently and reads that aligned uniquely were grouped into genomic sequence intervals of ~100 kb whereas reads that failed to align were binned with PE mates without *Eland* using the PE information. Reads that mapped in more than one location were discarded. To align binned reads to their respective 100 kb genomic sequence, *Crossmatch*, a Smith-Waterman-based local alignment algorithm was used based on the following parameters–minscore 21 and–masklevel 0 (http://www.phrap.org). Genotypes were identified using a Bayesian genotype caller, Most Probable Genotype [81]. Selected *de novo* variants detected exclusively by exome sequencing were tested by Sanger sequencing. Sanger sequencing was performed to confirm the segregation of the identified variant in *FOXR1* using the following primers 5’-AAAGCACTTCCCCTTTTTCC-3’ (forward) and 5’ AGTTGTTTGCCCATGGATTC-3’ (reverse).

### Construction of expression vectors

Full-length human pCMV-SPORT6 *FOXR1* plasmid was purchased from GE Dharmacon (clone ID 5164198; accession #BC038969). The human M280L variant in *FOXR1* was generated by introducing a point mutation at residue 280 (methionine to leucine) using QuikChange II Site-Directed Mutagenesis Kit (Agilent Technologies) in the pSport6 human FOXR1 plasmid with the following 5’-CCAACAGTGCTTGAGCCAGCCAG-3’ (forward) and 5’- ATACTTTCTAGCCGAGTGGAAG-3’ (reverse) primers and verified by nucleotide sequencing. The *FOXR1* wild-type and M280L mutant were then PCR amplified using 5′- AAAGCACTCGAGATGGGGAACGAGCTCTTTCTG-3’ (forward) and 5’-TTTGGCCCGCGGTTAAAGATCAAAGAGGAAGGG-3’ (reverse) primers and subcloned into the XhoI and SacII restriction sites of pEGFP-C3 (Clontech) to create an N-terminal EGFP tag. To generate the *FOXR1* C-terminal truncation variant, Δ280–292, we used full-length human GFP-tagged *FOXR1* wild-type as template and designed PCR 5’-AAAGCACTCGAGATGGGGAACGAGC-3’ (forward) and 5’-TTTGGCCCGCGGTTAGCACTGTTGGATACTTTCTAGCCG-3’ (reverse) primers to amplify the region encoding amino acids 1–279, which was subcloned into the XhoI and SacII restriction sites of pEGFP-C3.

### Cell culture

HEK293T (ATCC CRL-3216) and COS-7 (ATCC CRL-1651) cells were maintained in Dulbecco’s Modified Eagle’s Medium (DMEM) supplemented with 10% fetal bovine serum (FBS; Hyclone) and 1% Penicillin/Streptomycin in a 5% CO_2_ incubator at 37°C. Cells at 60% confluency were transfected with GFP, GFP-FOXR1 or GFP-M280L plasmids using FuGENE6 transfection reagent (Promega) according to the manufacturer’s instructions. For subcellular fractionation, cells were briefly washed with phosphate-buffered saline (PBS) and lysed in buffer A that consists of 50 mM Tris-HCl pH 7.5, 0.5% Triton-X100, 137.5 mM NaCl, 10% glycerol, 5 mM EDTA pH 8.0 with proteinase inhibitors. The lysate was centrifuged 850 x *g* for 15 min at 4°C. The supernatant “cytosolic” fraction was removed to a new tube and the remaining “nuclear” pellet was washed twice with buffer A at 4°C and centrifuged at 850 x *g* for 2 min. The pellet was then solubilized in buffer B that consists of 50 mM Tris-HCL pH 7.5, 0.5% Triton-X100, 137.5 mM NaCl, 10% glycerol, 5 mM EDTA pH 8.0, 0.5% SDS with proteinase inhibitors and sonicated for 5–10 secs. Equal amount of 2x sample buffer (0.1 M Tris-HCL, pH 6.8, 4% SDS, 20% glycerol, 10% β-mercaptoethanol, 0.01% bromophenol blue) was added to the tubes containing the nuclear and cytoplasmic fractions, boiled at 100°C for 10 min and subjected to SDS-PAGE. For MG132 treatment, transfected cells were treated with 50 μM MG132 (Sigma-Aldrich) for 24 h. Cells were then washed with PBS and solubilized in 2x sample buffer. Cellular stress paradigms: serum starvation, cells were incubated in DMEM without fetal bovine serum for 24 h at 37°C; for CO_2_ deprivation, cells were deprived of 5% CO_2_ for 24 h at 37°C; PMA treatment, cells were treated with 1 μM of phorbol 12-myristate 13-acetate (PMA, Sigma) for 24 h at 37°C.

### Western blotting

Whole cell lysates were extracted from cells in 2x sample buffer and separated on 10% SDS–PAGE gel and transferred to nitrocellulose membranes (GE Healthcare). The membranes were blocked with Odyssey Blocking Buffer in PBS (Licor), followed by incubation with primary antibodies against human FOXR1 (Biorbyt, rabbit 1:200), GFP (synaptic systems, mouse 1:1000), GAPDH (EMD Millipore, mouse 1:5000), HSPA1A and HSPA6 (Enzo life sciences, mouse 1:1000), DHRS2 (Abcam, rabbit 1:500), Histone H3 (Cell-Signaling, rabbit 1:1000) overnight at 4°C. Proteins recognized by the antibodies were detected with an Odyssey infrared imaging system (LI-COR) using IRDye680RD- or IRDye800CW-coupled secondary antibodies (LI-COR, 1: 20,000).

### Immunocytochemistry and image analysis

Transfected cells plated on coverslips were washed briefly with PBS and fixed with 4% paraformaldehyde at room temperature for 10 min, permeabilized and blocked in 10% goat serum, 0.1% saponin in PBS. To detect oxidative stress following PMA treatment, transfected cells were incubated with 5 μM CellROX Oxidative Stress Reagent (Thermo Fisher Scientific) for 30 min at 37°C prior to fixation with paraformaldehyde. To detect aggresomes in transfected cells, we used the PROTEOSTAT Aggresome Detection Kit (Enzo Life Sciences). The dye intercalates into the cross-beta spine of quaternary protein structures found in misfolded and aggregated proteins. Coverslips were mounted with ProLong Gold Anti-Fade Mount with DAPI (Fisher Scientific) and imaged with a Carl Zeiss LSM700 confocal microscope. Images were collected with identical confocal settings for all of the samples and Z-stacked images were projected with maximal projection mode using Zeiss Confocal Software.

### RNA sequencing and analysis

HEK293T were transfected with GFP, GFP-FOXR1 or GFP-M280L mutant using FuGENE6. Forty-eight hours after transfection, total RNA was purified using the QIAshredder and RNeasy Mini Kit (QIAGEN) and samples were processed with Trizol (Invitrogen). Three biological replicates were processed independently. RNA samples were suspended in DEPC-treated water and concentrations were determined using the Nanodrop ND-1000 (Thermo Scientific) where all samples showed A260/A280 ratios higher than 2.0. RNA integrity was also checked in a bioanalyzer (Agilent 2100). Library preparations and sequencing were performed by The Broad Institute, Cambridge, MA using Illumina HiSeq 2000 technology.

The RNA sequencing reads were aligned to the GRCh38 *Homo sapiens* genome using HISAT2 [82] with default parameters. The bam files were sorted by read names instead of chromosome coordinates by SAMtools [83]. Gene count matrix of each sample was generated by HTSeq, a Python framework to work with high-throughput sequencing data [84]. Downstream analysis was performed with the DEseq2 [85] package in R. Genes that were not expressed in any cell were removed from downstream analysis. Sample PCA plots were generated with ‘plotPCA’ function to detect and remove the outlier sample(s) in each condition. Differential expression analysis between conditions was performed with the ‘DESeq’ function with default parameters. Log-fold change shrinkage was performed on the differential expression analysis result. DEGs with adjust-p value < 0.05 and log-fold-change > 0.25 were kept for downstream analysis. Heatmaps of DEGs were visualized with heatmap and genes with similar expression patterns across samples were clustered on the heatmap. Gene set enrichment analysis was conducted with the GSEA [86]. Gene ontology and enrichment analysis encompassing the DEGs were analyzed using the Database for Annotation, Visualization, and Integrated Discovery (DAVID v6.8) software where the threshold was set as modified Fisher Exact *P*-value (EASE score) ≤ 0.05.

The package HOMER [87] was used to find the FOXR1 binding motif in the promotor region of DEGs. The method findMotifs.pl was used with the -find option. The input of the analysis included: the gene symbols of genes in each cluster of DEG result except cluster 4, which has little amount of DEGs and the motif matrix files which contains the forward and reverse-backward sequence of primary and secondary binding motif of FOXR1. The percentage of DEGs with FOXR1 binding motif in the promotor regions was summarized using in-house R script.

### Quantitative real-time PCR analyses

Total RNA was purified using the QIAshredder and RNeasy Mini Kit (Qiagen). The cDNA was synthesized using iScript cDNA Synthesis Kit (Biorad) or Accuris qMax cDNA Synthesis Kit (Midland Scientific). Quantitative real-time PCR was performed in an ABI Prism 7900HT Fast Real-Time PCR System (Applied Biosystems) using the Power SYBR Green PCR Master mix (Thermo Scientific) with a two-step cycling protocol and an annealing/extension temperature of 60°C. The experiment was performed with three biological replicates and three technical replicates each. The relative amount for each target was normalized using GAPDH or 18s as a reference gene and the fold change in gene expression was calculated using the ΔΔCt method with the GFP-transfected cells serving as control. Primers were as follows, human *DHRS2*: 5′-TCATCAGCTGCAGAGGATTGG-3′ (forward) and 5′-AATGTTCTCCCCGTTGACGTA-3′ (reverse); human *DNAJC6*: 5’-AGGACAACTTGAAAGACACCCT-3’ (forward) and 5’-AAATCTCCCTTTGTGTAGCTGG-3’ (reverse); human *DNAJC21*: 5’-CCTGAAATGGCACCCGGATAA-3’ (forward) and 5’-TTTCCTGAGGGTCACTCAACA-3’ (reverse); human *GAPDH*: 5′-GGATTTGGTCGTATTGGG-3′ (forward) and 5′-GGAAGATGGTGATGGGATT-3′ (reverse); human *HSPA1A*: 5′-GCCTTTCCAAGATTGCTGGTT-3′ (forward) and 5′-TCAACATTGCAAACACAGGA-3′ (reverse); human *HSPA6*: 5′-CAAGGTGCGCGTATGCTAC-3′ (forward) and 5′-GCTCATTGATGATCCGCAACAC-3′ (reverse); human *HSPA12A*: 5’- GCTCCCACATCTGCATATTCAT-3’ (forward) and 5’-TTCTGAGACGTTGGAGTCAGT-3’ (reverse); human *SACS*: 5’-ACAACAACGCGGTTTTCACC-3’ (forward) and 5’- GCCTGATTCATGTGGGCCAA-3’ (reverse). mouse *Foxr1*: 5’- GATGGTCCAGACATTAAGCCC-3’ (forward) and 5’-GCTGCTGTACCTCCGAAGC-3’ (reverse). mouse *18S*: 5’-CGAACGTCTGCCCTATCAACT-3’ (forward) and 5’- CTGCCTTCCTTGGATGTGGT -3’ (reverse). Data analysis was performed using the ABI Prism 7900HT SDS Software.

### Dual luciferase assay

The FOXR1 DNA-binding motif was located by analyzing 3000 kb of the upstream regulatory sequences of human *HSPA6*, *HSPA1A*, *DHRS2*. These regions were amplified by PCR from genomic DNA isolated from HEK293T cells (Genomic DNA purification kit, Thermo Fisher Scientific) using the following primer sets for human *HSPA6*: 5’- TTCT*GGTACC*CACCGGGCCTCTGGAGACG -3’ (forward) and 5’- TTCT*GCTAGC*CGGATCTGCTCAGCTCCGC-3’ (reverse); human *HSPA1A*: 5’- TTCT*GGTACC*GGCTGCTCCGACCAATCAATC-3’ (forward) and 5’- GCTCCTCAG*GCTAGC*CGTTATC-3’ (reverse) and subcloned into the KpnI-NheI sites of pNL3.1[Nluc/minP] reporter plasmid (Promega). For the human *DHRS2*: 5’-TGCAGGTGCCAGAACATTTCTCTTAATGCCAAATCATTTCCCAAAGTGATTGTACTTACC (forward) and 5’-TGGTGGCTTTACCAACAGTACCGGATTGCCAGAGTTGTTCATTCCTCTCGGTGCATTC-3’ (reverse) and Gibson cloned into pNL3.1[Nluc/minP]. HEK293T cells were transfected using FuGENE6 transfection reagent (Promega) with the normalization plasmid pGL4.54[Luc2/TK] (Promega), the respective reporter plasmid (pNL3.1-Nluc/minP-HSPA1A, pNL3.1-Nluc/minP-HSPA6 or pNL3.1-Nluc/minP-DHRS2) and GFP, GFP-tagged FOXR1 or M280L expression plasmids. Transfected cells were collected in PBS and luciferase activity was assessed using the Nano-Glo Dual Luciferase reporter assay system (Promega). Dual luciferase signal was quantified using a VICTOR-3 plate reader (Perkin Elmer). To control for transfection efficiency, the Nluc reporter plasmid signal was normalized to the constitutive luciferase signal (i.e., signal from pGL4.54[Luc2/TK] plasmid, Nluc/Luc2). Fold-induction values for each upstream gene regulatory sequence were calculated relative to the background activity of reporter plasmid in the presence of GFP-FOXR1 or GFP-M280L plasmid. Reporter assays were performed as three biological replicates with three technical replicates per biological replicate.

For the HSF1 reporter assay, the upstream transcriptional regulatory region of human FOXR1 containing the HSF1 binding motif (TTCTAGAA) was amplified by PCR from genomic DNA isolated from HEK293T cells (Genomic DNA purification kit, Thermo Fisher Scientific) using the following primer set, human *FOXR1*: 5’-TTCT*GGTACC*GTCCCCCAGGCTGGAG-3’ (forward) and 5’-GCCAGGACTTCTCTAATTTCCGCAG*CGATCG*TCTT-3’ (reverse) and subcloned into the KpnI-NheI sites of pNL3.1[Nluc/minP] reporter plasmid (Promega). A second pNL3.1-Nluc/minP-FOXR1 reporter plasmid was generated by disrupting the HSF1 binding motif (mutating to GGCTAGAA termed Mut) with the following primers for human *FOXR1*: 5’- CTTTCATAACGGCTAGAAAGTAACTACTAATAC-3’ (forward) and 5’- GTTTATGGTTTATCCACG-3’ (reverse) using Q5 Site-Directed Mutagenesis Kit (New England Biolabs) and verified by nucleotide sequencing. HEK293T cells were transfected using FuGENE6 transfection reagent (Promega) with the normalization plasmid pGL4.54-LuPrc2/TK, the respective reporter plasmid (pNL3.1-Nluc/minP-FOXR1-WT or pNL3.1-Nluc/minP-FOXR1-Mut) and GFP control or GFP-tagged HSF1 expression plasmids (32538, Addgene). Transfected cells were collected and analyzed as detailed above.

### Lactate dehydrogenase assay

HEK293T cells were transfected with GFP, GFP-FOXR1 or GFP-M280L plasmids using FuGENE6 transfection reagent (Promega). Cells were treated with 1 μM PMA (Sigma) for 24 hours and lactate dehydrogenase (LDH) cytotoxicity assay (Thermo Fisher Scientific) was performed from the media collected according to manufacturer’s instructions. As a positive control, one set of cells were treated with the positive control lysis buffer provided with the kit. The 490 nm readout was measured in a colorimetric plate reader (BioRad).

### Detergent extraction assay

Transfected HEK293T cells were isolated in 50 mM Tris-HCl buffer at pH 7.5 with protease inhibitors by brief sonication and centrifuged at 350,000 x *g* for 15 min and the supernatant was collected as a Tris-HCl soluble fraction [adapted from 88]. The resulting pellet was sequentially extracted in Tris-HCl buffer containing 1% Triton X-100, then by 1% Sarkosyl and finally by 2% SDS. Each detergent extraction step was incubated for 1 h at 4°C and ultracentrifgued at 350,000 x *g* for 15 min, resulting in a Triton X-100 soluble fraction, Sarkosyl soluble fraction and SDS soluble fraction, respectively. The Tris-HCl fraction containing 20 μg of total proteins, along with equal volumes of Triton X-100, Sarkosyl and SDS fractions were loaded onto SDS-PAGE.

### Gene editing with CRISPR/Cas9 to generate *Foxr1* mouse knockout

The *Foxr1* (*C57BL/6N-Foxr1<em1(IMPC)Tcp>*) mouse line was made as part of the KOMP2-Phase2project at The Centre for Phenogenomics, Canada and obtained from the Canadian Mouse Mutant Repository. The *Foxr1* knockout mouse line was generated by injecting Cas9 ribonucleoprotein complexes and single guide RNAs (gRNAs) with spacer sequences of GGCCAAGCCCGGGTAGTATG and TCCACTGTTACCCCATGATC targeting the 5’ side and CCGCAAGCCATCAGCCCAGA and TGAGTGCCAAGGCAATCAGA targeting the 3’ side. This leads to a deletion of exons 2, 3 and majority of exon 4, thus destroying exon 4 splice acceptor that results in a 976 bp deletion from Chr9:44435486 to 44436461, leading to a frameshift mutation in the *Foxr1* full-length protein coding transcript.

### PCR genotyping

Genomic DNA from tails was isolated to determine the wild-type and deleted *Foxr1* alleles. Primer set 1 to detect a 354 bp product for wild type and heterozygotes were *Foxr1* 5’- CCACAGCTCTGCCATATAGACTAG-3’ (forward) and 5’- GAGAGAGAAGAGTCAAGAGAAAGGC-3’ (reverse). Primer set 2 to detect 403 bp and 1381 bp products were *Foxr1* 5’- CCACAGCTCTGCCATATAGACTAG-3’ (forward) and 5’- GGGTAGGAGTGGTTATGTTCTTGAG-3’ (reverse). The larger 1381 bp band represents *Foxr1* wild-type; both 403 bp and 1381 bp bands represent heterozygotes and only the 403 bp band is present in homozygotes.

### Brain histology and image analysis

Neonatal mouse pups of both sexes from breeding pairs of heterozygous *Foxr1* mutant mice were collected and terminated by using a sterile sharp scissor to remove the head. The brains were immediately removed and fixed in 4% paraformaldehyde and 5% sucrose in PBS at 4°C overnight. Brains were then transferred to 10% sucrose in PBS at 4°C for 24 h, followed by 24 h in 20% sucrose in PBS, then 24 h in 30% sucrose at 4°C. Serial sucrose to OCT compound dilutions were performed as follows: brains were incubated in a 2:1 mixture of 30% sucrose:OCT for 25 min with gentle rocking at room temperature, followed by 25 min in 1:1, and 25 min in 1:2 30% sucrose:OCT. Brains were placed in tissue molds with OCT compound and allowed to rest another 20 min prior to freezing in 2-methylbutane with dry ice and stored at -80°C until ready to use. Brains were sectioned at 20 μm using a LEICA CM1850 cryostat (LEICA Biosystems) and mounted on SuperFrost microscope slides (Fisher Scientific) and kept at -20°C.

Prior to Nissl staining, sections were removed from -20°C and allowed to dry for ~6 h at room temperature. Slides were stained with 1% cresyl violet solution (Sigma C-1791) for 2.5 min. Slides were then incubated in the following series of solutions for 5 s each: ddH_2_O, ddH_2_O, 50% EtOH, 70% EtOH, and 95% EtOH, 100% EtOH and cleared in xylene (Fisher X4-4). Slides were mounted with Permount media (Fisher SP15-100) and dried overnight before imaging. For immunostaining, brain sections were allowed to warm to room temperature before being rehydrated and washed with PBS for 20 min. HistoVT One (Nacalai USA) antigen retrieval solution was prewarmed to 70°C. PBS was replaced with antigen retrieval solution and sections were incubated for 20 min in 70°C water bath, followed by 15 min cooling on benchtop. Sections were washed in PBS and incubated with blocking solution (5% goat serum, 0.3% Triton X-100 in PBS) for 1 h at room temperature. Brain sections were then incubated with primary polyclonal rabbit anti-MAP2 antibody (1:500 dilution in 5% goat serum in PBS; cat# AB5622 Millipore) at 4°C overnight. The next day, brain sections were washed in PBS and incubated in secondary antibody Alexa Fluor 488 goat anti-rabbit IgG (1:500 dilution in 5% goat serum in PBS; cat# AB_2576217 ThermoFisher) for 1 h at room temperature. After final wash, slides were mounted using Prolong Gold with DAPI (cat# P-36931 Fisher). Images were captured using a Zeiss LSM 700 confocal microscope using the 25x objective, pixel size 0.5 μm, with correction collar set to oil immersion. Nissl stain sections were captured in brightfield. MAP2 staining was detected with 488 nm excitation and DAPI was detected with 405 nm excitation. Images were captured in a single focal plane using a bounding grid tile scan and images were stitched together post-acquisition. Images were then imported to FIJI (ImageJ) where the line tool was used to measure cortical thickness in microns from the pia to the corpus callosum at 0°, 45°, and 90° relative to the midline. The freehand selection tool was used to trace the borders of ventricles, from which ventricle cross-sectional area was calculated.

### Statistical analysis

To determine statistical significance, we used either a Student’s *t*-test to compare two groups, or a one-way analysis of variance (ANOVA) with post hoc Tukey’s test for multiple comparison. All bars and error bars represent the mean ± S.E.M. and significance was set at p<0.05. The data were analyzed using GraphPad Prism 7 software (GraphPad Software Inc., San Diego, CA). All reported data for this study are listed in S1 Data

## Supporting information

S1 Table Summary of clinical features of the proband with a *de novo* variant in *FOXR1*. (DOCX)

S1 Fig Residue alignment of FOXR1 across species. C-terminal amino acid sequence shows the conserved methionine residue (indicated in red) within a highly conserved region of FOXR1. Numbers indicate amino acid residues.
(TIF)

S2 Fig The M280L variant induces nuclear puncta phenotype in COS7 cells. Fluorescence images of COS7 cells transfected with GFP or GFP-tagged plasmids of human FOXR1 WT or M280L. DAPI (blue) served as a nuclear marker. Scale bar = 20 μm.
(TIF)

S3 Fig RNAseq analysis. **(A)** PCA plot of the three groups clustered separately in multidimensional scaling analyses. Groups of samples analyzed using Principal Component Analysis (PCA) plots where replicates are clustered together and clusters from different conditions are separated. **(B)** Pie chart showing the distribution of 2644 differentially-expressed genes between GFP versus FOXR1 WT and 735 differentially-expressed genes between FOXR1 WT versus M280L. **(C)** Pearson’s correlation plot examining log2 (fold change) between FOXR1 WT with GFP and M280L with GFP. **(D)** Table of the percentage of differentially expressed genes (DEGs) that have FOXR1 consensus sequence from clusters A, B, D, and E.
(TIF)

S4 Fig Gene ontology (GO) enrichment analysis between FOXR1 WT and M280L and GFP control and FOXR1 WT. Normalized enrichment scores indicate the distribution of biological processes across a list of genes ranked by hypergeometrical score. Higher enrichment scores indicate a shift of genes belonging to certain GO categories towards either end of the ranked list, representing up or down-regulation (positive or negative values, respectively). **(A)** GO enrichment analysis between WT and M280L that is downregulated in M280L. **(B)** GO enrichment analysis between WT and M280L that is upregulated in M280L. **(C)** GO enrichment analysis between GFP and WT that is downregulated in WT. **(D)** GO enrichment analysis between GFP and WT that is upregulated in WT. **(E)** GO enrichment analysis between GFP and M280L that is downregulated in M280L. **(F)** GO enrichment analysis between GFP and M280L that is upregulated in M280L.
(TIF)

S5 Fig Promoter sequences showing *FOXR1* response elements lie upstream of the HSEs in both the *HSPA6* and *HSPA1A* promoters. In addition, we identified a consensus sequence for binding by HSF1 within the promoter region of *FOXR1*.
(DOCX)

S6 Fig Mouse *Foxr1* expression and knockout strategy by CRISPR/Cas9 gene editing. **(A)** Amino acid sequence shows human FOXR1 shares 66% amino acid sequence identity with its mouse homologue. Numbers indicate amino acid residues. **(B)** qPCR using specific primer-targeting mouse *Foxr1* shows *Foxr1* expression in several tissues, including heart, liver, lung and high expression in the brain at embryonic day 17. **(C)** Sanger sequencing analyses illustrates the two gRNAs (indicated in red) used to generate *Foxr1* knockout mice (top) and confirms the 979 bp deletion (bottom). Dashed lines in *Foxr1* wild-type allele represents protospace between the two gRNAs. Dashed line boxed in red in *Foxr1* knockout allele indicate the 979 bp deletion.
(TIF)

S7 Fig Quantifications of cortical thickness and ventricle size in *Foxr1* wild-type and knockout mice. **(A-D)** Representative images and quantification of brain sections anterior to bregma of 4 wild-type and 4 *Foxr1* knockout mice at 0°, 45° and 90° (relative to the midline) to pia surface, respectively. Graph represents relative thickness normalized to wild-type (WT). Unpaired *t*-test (0°, p = 0.0021; 45°, p = 0.0054; 90°, p = 0.2369). **(E)** Graph of ventricle area from brain sections anterior to bregma. Unpaired *t*-test p = 0.0405. **(F-I)** Representative images and quantification of brain sections at bregma of 4 wild-type and 4 *Foxr1* knockout mice at 0°, 45° and 90° (relative to the midline) to pia surface, respectively. Graph represents relative thickness normalized to wild-type (WT). Unpaired *t*-test (0°, p = 0.3208; 45°, p = 0.0447; 90°, p = 0.0368). **(J)** Graph of ventricle area from brain sections at bregma. Unpaired *t*-test p = 0.2049. **(K-N)** Representative images and quantification of brain sections posterior to bregma of 4 wild-type and 4 *Foxr1* knockout mice at 0°, 45° and 90° (relative to the midline) to pia surface, respectively. Graph represents relative thickness normalized to wild-type (WT). Unpaired *t*-test (0°, p = 0.0745; 45°, p = 0.0811; 90°, p = 0.0253). **(O)** Graph of ventricle area from brain sections posterior to bregma. Unpaired *t*-test p = 0.040.
(TIF)

S1 Video The M280L mutant exhibits nuclear puncta phenotype in response to CO_2_ stress. Time-lapse video of M280L in response to CO_2_ stress in transfected HEK293T cells.
(MP4)

S2 Video The M280L mutant exhibits nuclear puncta phenotype in response to PMA treatment. Time-lapse video of M280L in response to PMA treatment.
(WMV)

S3 Video The M280L mutant exhibits nuclear aggregates in response to PMA treatment. Time-lapse video of M280L in response to PMA treatment at high magnification.
(AVI)

S1 Data All reported data for this study. (XLSX)
