# Supplementary material for: FOXR1 regulates stress response pathways and is necessary for proper brain development
Source: PLoS Genet. 2021 Nov 1;17(11):e1009854. doi: 10.1371/journal.pgen.1009854 (PMC8559929; doi:10.1371/journal.pgen.1009854)
Supplement: S1 Table — (DOCX) [file pgen.1009854.s001.docx]

| Neuro Phenotypes | Cerebellar Abnormalities | Physical Abnormalities | Facial Abnormalities | Muscle  Abnormalities | Visual  Abnormalities | Other |
| --- | --- | --- | --- | --- | --- | --- |
| Global developmental delays | Cerebellar hypoplasia | Hip dysplasia | Myopathic facies | Hyperactive deep tendon reflexes | Optic atrophy | Exaggerated startle response |
| Postnatal Microcephaly | Abnormality of the cerebellar penduncle | Growth Delay | Preauricular pit | Joint hypermobility | Cortical visual impairment | Aspiration |
| Cerebral white matter atrophy | Cerebellar atrophy | Decreased body weight | Anteverted nares | Severe muscle hypotonia | Retinitis pigmentosa | Constipation |
| Abnormality of midbrain morphology | Aplasia/Hypoplasia of the cerebellum | Short stature | Low set ears | Generalized hypotonia/  dystonia |  |  |
| Abnormality of the medulla oblongata |  | Ankle Clonus |  | Muscular hypotonia of the trunk |  |  |
| Dandy –Walker malformation |  | Bell-shaped thorax |  | Poor head control |  |  |
| Hypoplasia of the pons |  | Bilateral single transverse palmar creases |  | Decreased fetal movement |  |  |
| Neuronal loss in the cerebral cortex |  | Scoliosis |  |  |  |  |
| EMG: Neuropathic changes |  |  |  |  |  |  |
| EEG with generalized slow activity |  |  |  |  |  |  |
| Low CSF 5-methyltetrahydro-folate |  |  |  |  |  |  |
| Dilation of lateral ventricle |  |  |  |  |  |  |
| Diffuse cerebral atrophy |  |  |  |  |  |  |
