# Supplementary figures and images for: FOXR1 regulates stress response pathways and is necessary for proper brain development
Source: PLoS Genet. 2021 Nov 1;17(11):e1009854. doi: 10.1371/journal.pgen.1009854 (PMC8559929; doi:10.1371/journal.pgen.1009854)

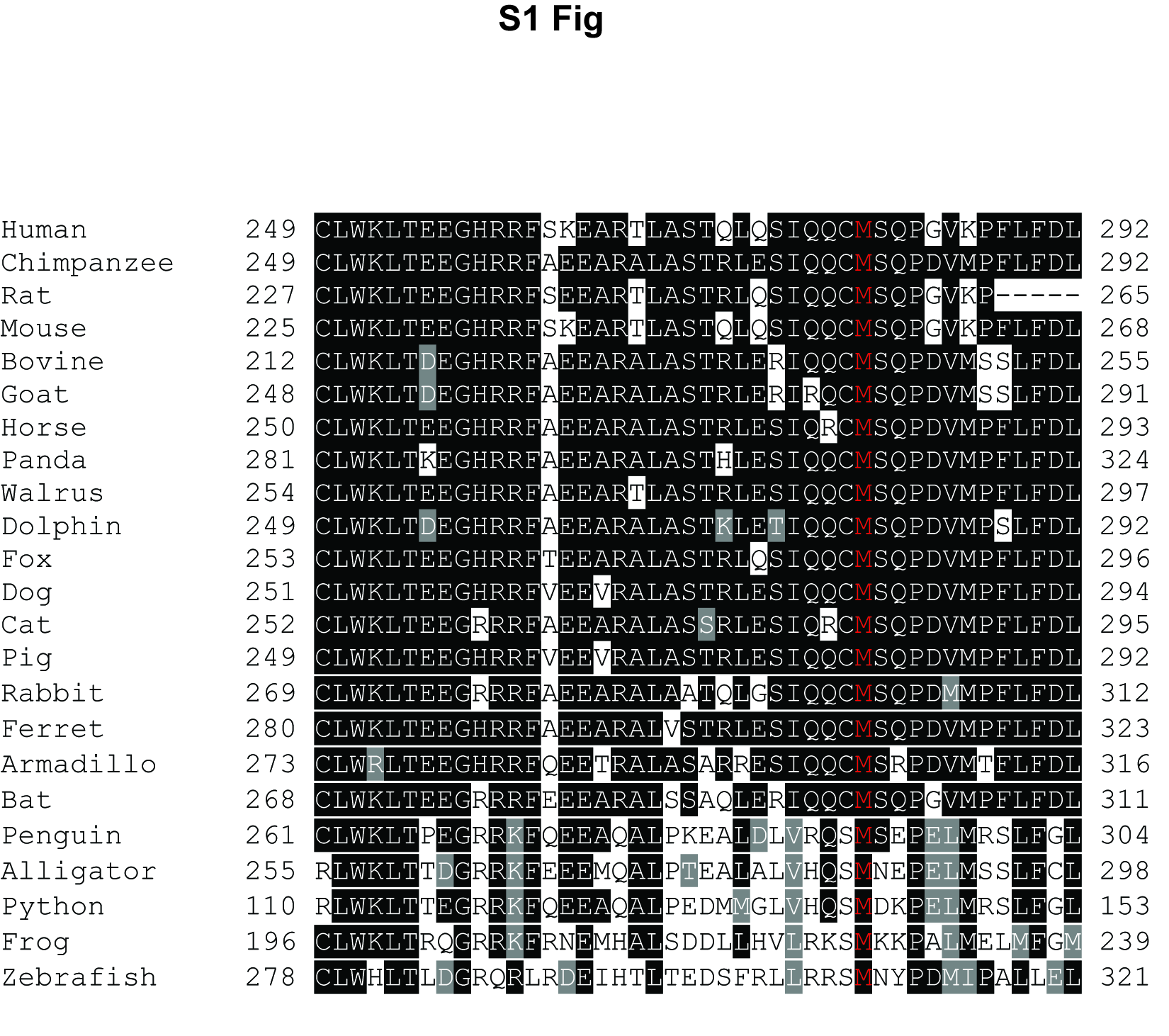

Supplement: S1 Fig — C-terminal amino acid sequence shows the conserved methionine residue (indicated in red) within a highly conserved region of FOXR1. Numbers indicate amino acid residues. (TIF) [file pgen.1009854.s002.tif]

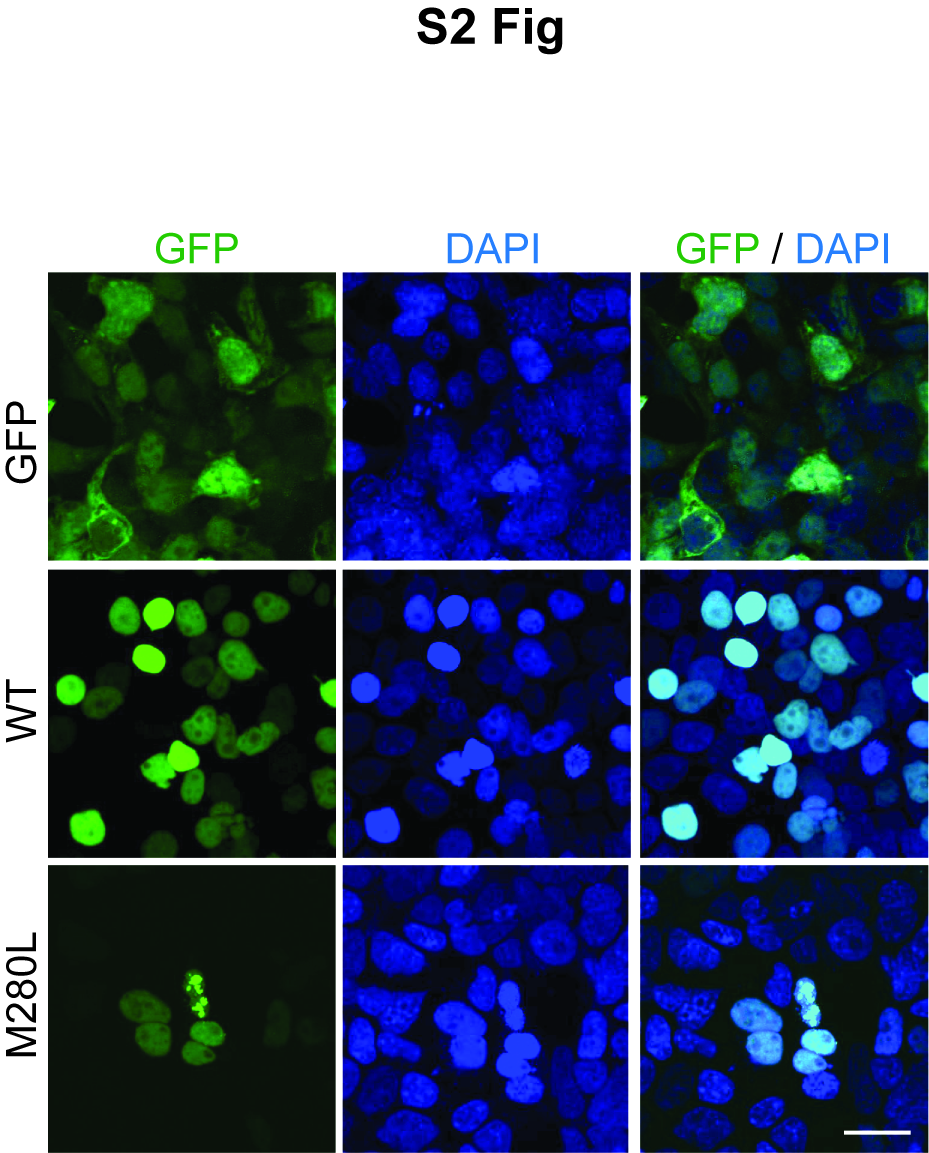

Supplement: S2 Fig — Fluorescence images of COS7 cells transfected with GFP or GFP-tagged plasmids of human FOXR1 WT or M280L. DAPI (blue) served as a nuclear marker. Scale bar = 20 μm. (TIF) [file pgen.1009854.s003.tif]

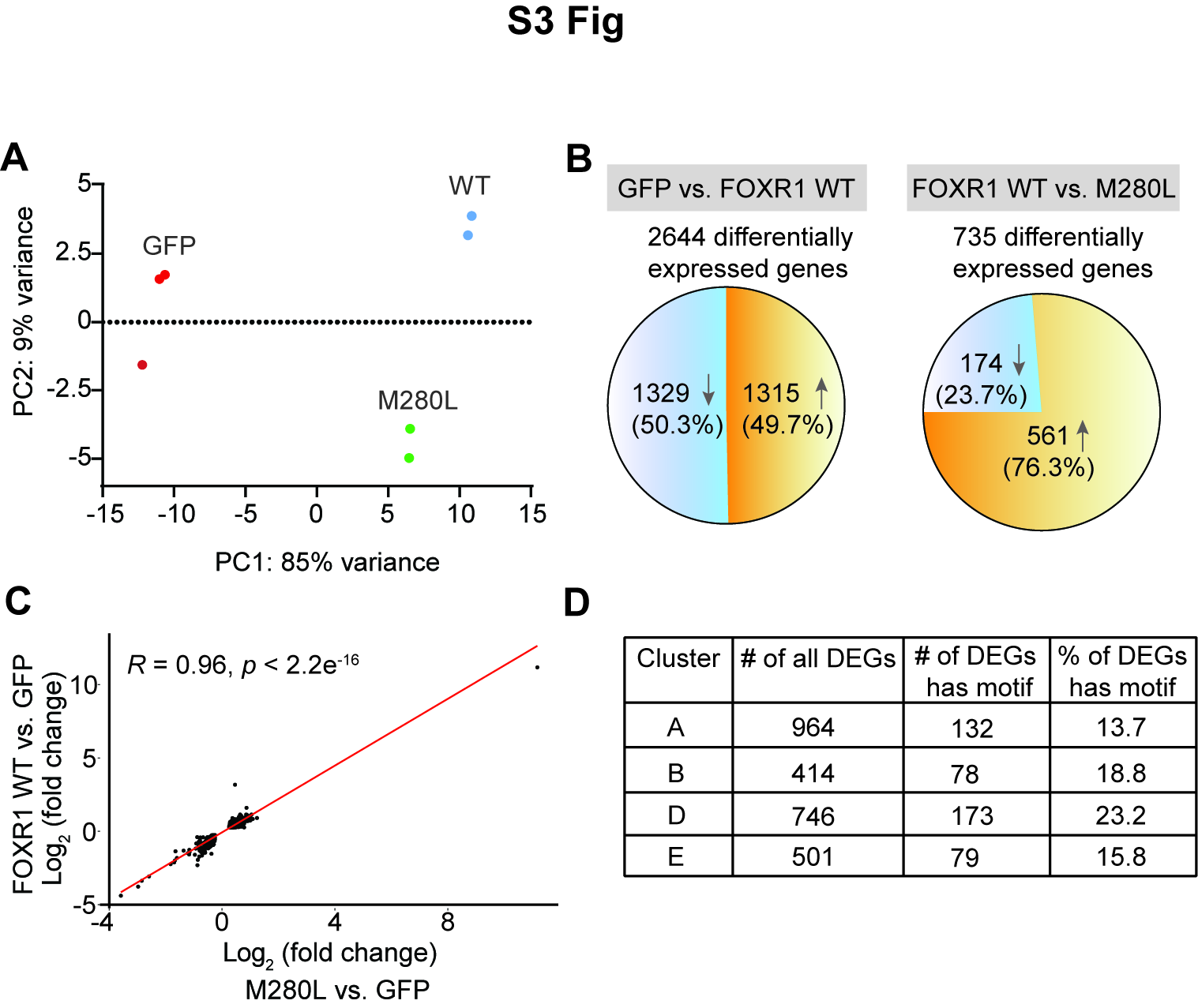

Supplement: S3 Fig — (A) PCA plot of the three groups clustered separately in multidimensional scaling analyses. Groups of samples analyzed using Principal Component Analysis (PCA) plots where replicates are clustered together and clusters from different conditions are separated. (B) Pie chart showing the distribution of 2644 differentially-expressed genes between GFP versus FOXR1 WT and 735 differentially-expressed genes between FOXR1 WT versus M280L. (C) Pearson’s correlation plot examining log2 (fold change) between FOXR1 WT with GFP and M280L with GFP. (D) Table of the percentage of differentially expressed genes (DEGs) that have FOXR1 consensus sequence from clusters A, B, D, and E. (TIF) [file pgen.1009854.s004.tif]

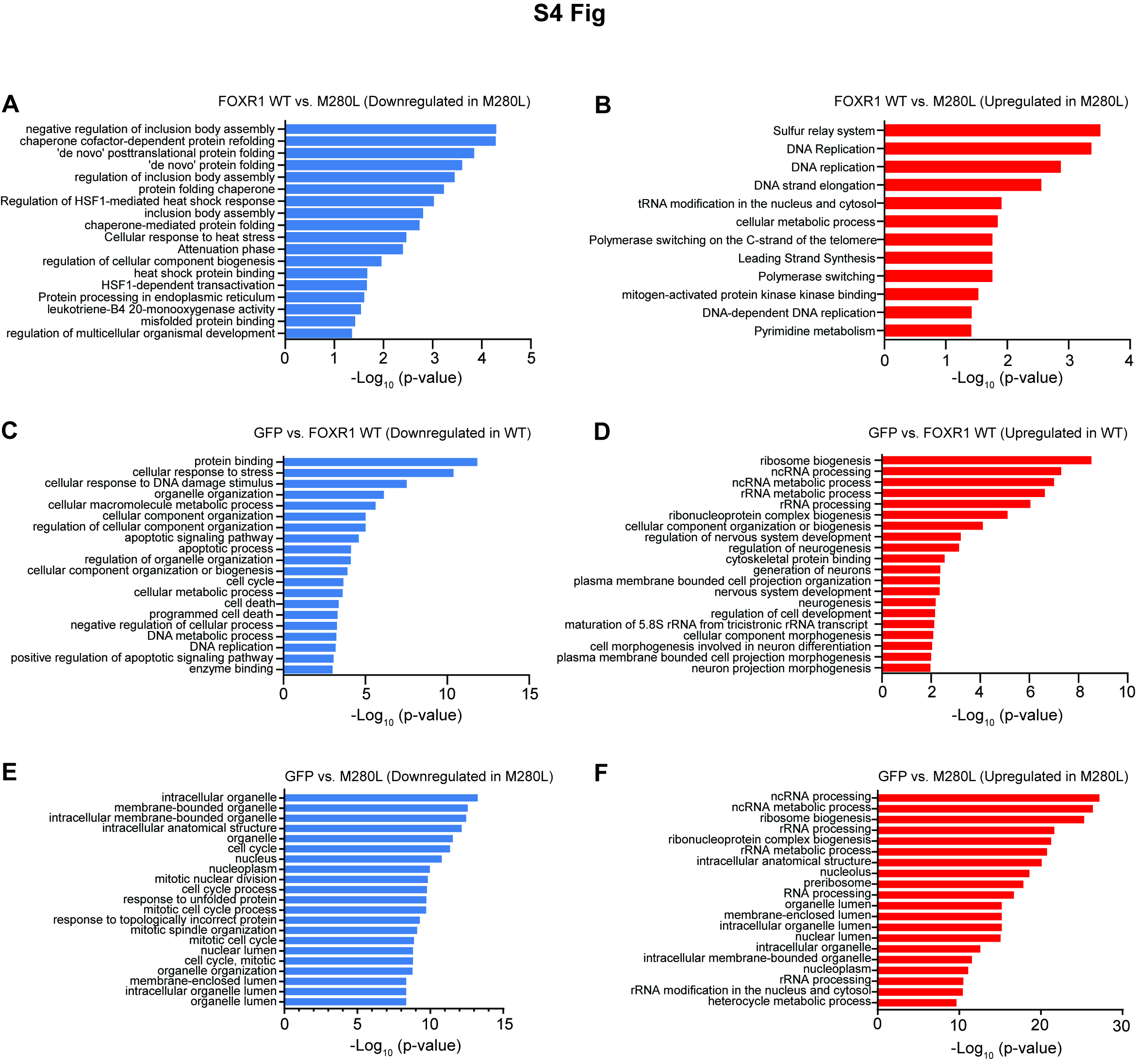

Supplement: S4 Fig — Normalized enrichment scores indicate the distribution of biological processes across a list of genes ranked by hypergeometrical score. Higher enrichment scores indicate a shift of genes belonging to certain GO categories towards either end of the ranked list, representing up or down-regulation (positive or negative values, respectively). (A) GO enrichment analysis between WT and M280L that is downregulated in M280L. (B) GO enrichment analysis between WT and M280L that is upregulated in M280L. (C) GO enrichment analysis between GFP and WT that is downregulated in WT. (D) GO enrichment analysis between GFP and WT that is upregulated in WT. (E) GO enrichment analysis between GFP and M280L that is downregulated in M280L. (F) GO enrichment analysis between GFP and M280L that is upregulated in M280L. (TIF) [file pgen.1009854.s005.tif]

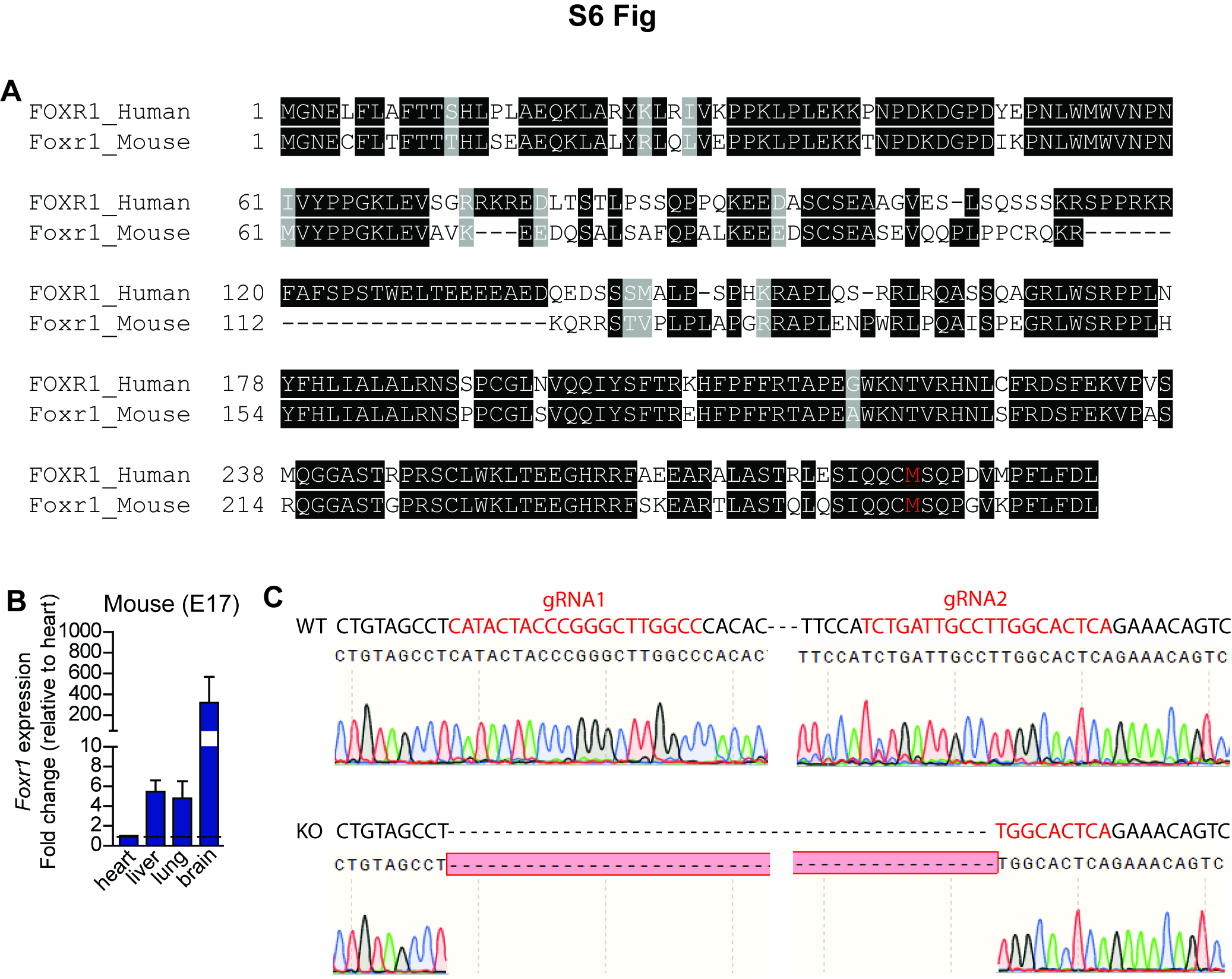

Supplement: S6 Fig — (A) Amino acid sequence shows human FOXR1 shares 66% amino acid sequence identity with its mouse homologue. Numbers indicate amino acid residues. (B) qPCR using specific primer-targeting mouse Foxr1 shows Foxr1 expression in several tissues, including heart, liver, lung and high expression in the brain at embryonic day 17. (C) Sanger sequencing analyses illustrates the two gRNAs (indicated in red) used to generate Foxr1 knockout mice (top) and confirms the 979 bp deletion (bottom). Dashed lines in Foxr1 wild-type allele represents protospace between the two gRNAs. Dashed line boxed in red in Foxr1 knockout allele indicate the 979 bp deletion. (TIF) [file pgen.1009854.s007.tif]

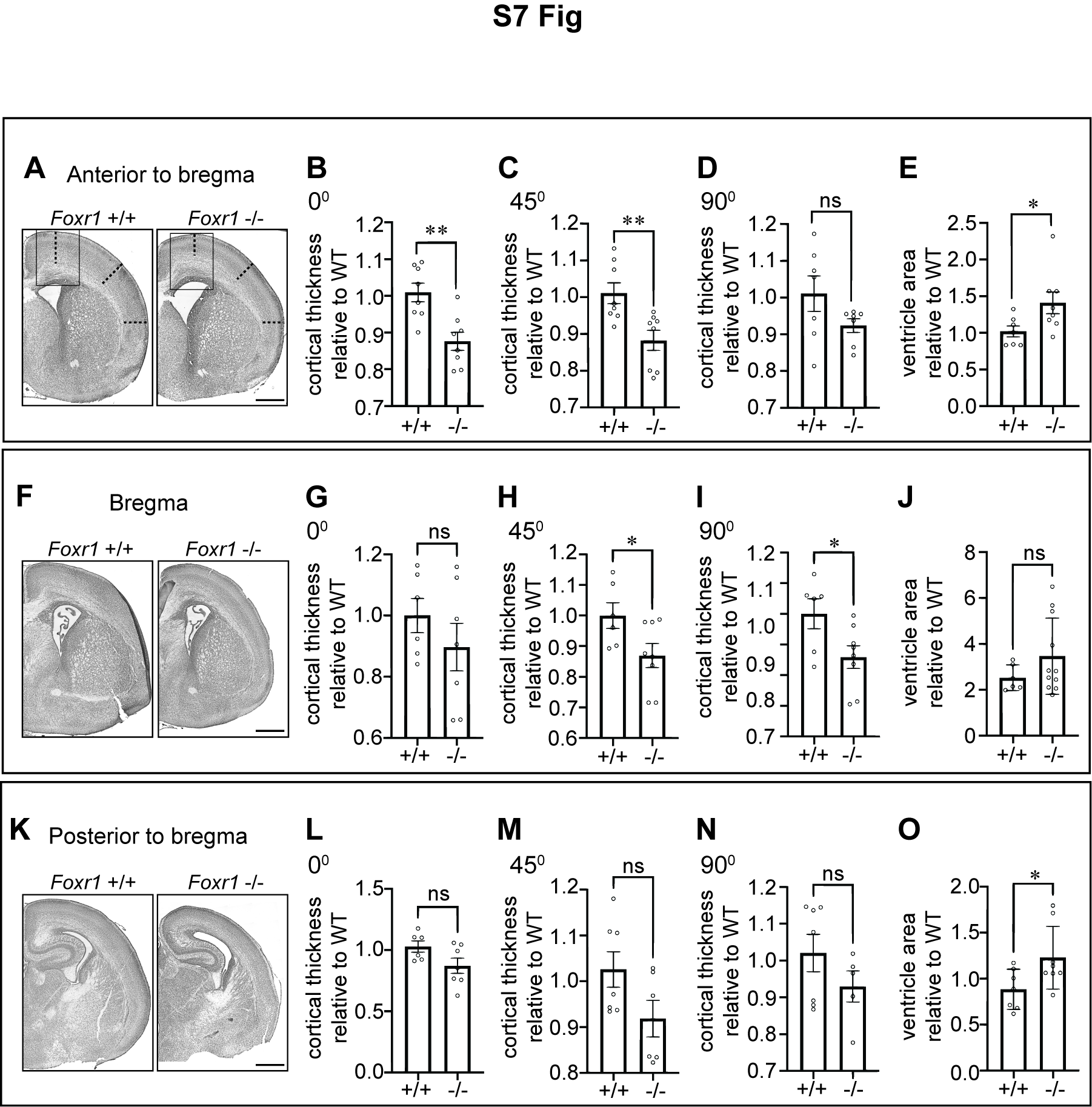

Supplement: S7 Fig — (A-D) Representative images and quantification of brain sections anterior to bregma of 4 wild-type and 4 Foxr1 knockout mice at 0°, 45° and 90° (relative to the midline) to pia surface, respectively. Graph represents relative thickness normalized to wild-type (WT). Unpaired t-test (0°, p = 0.0021; 45°, p = 0.0054; 90°, p = 0.2369). (E) Graph of ventricle area from brain sections anterior to bregma. Unpaired t-test p = 0.0405. (F-I) Representative images and quantification of brain sections at bregma of 4 wild-type and 4 Foxr1 knockout mice at 0°, 45° and 90° (relative to the midline) to pia surface, respectively. Graph represents relative thickness normalized to wild-type (WT). Unpaired t-test (0°, p = 0.3208; 45°, p = 0.0447; 90°, p = 0.0368). (J) Graph of ventricle area from brain sections at bregma. Unpaired t-test p = 0.2049. (K-N) Representative images and quantification of brain sections posterior to bregma of 4 wild-type and 4 Foxr1 knockout mice at 0°, 45° and 90° (relative to the midline) to pia surface, respectively. Graph represents relative thickness normalized to wild-type (WT). Unpaired t-test (0°, p = 0.0745; 45°, p = 0.0811; 90°, p = 0.0253). (O) Graph of ventricle area from brain sections posterior to bregma. Unpaired t-test p = 0.040. (TIF) [file pgen.1009854.s008.tif]
