# Supplementary material for: FOXR1 regulates stress response pathways and is necessary for proper brain development
Source: PLoS Genet. 2021 Nov 1;17(11):e1009854. doi: 10.1371/journal.pgen.1009854 (PMC8559929; doi:10.1371/journal.pgen.1009854)
Supplement: S5 Fig — In addition, we identified a consensus sequence for binding by HSF1 within the promoter region of FOXR1. (DOCX) [file pgen.1009854.s006.docx]

**S5 Fig**

FOXR1 Binding site (Primary CAAACA/ Partial GACGCC)

HSEs

NNNNN Ares not cloned

**HSPA1A Promoter (-1043 🡪-152)**

TCCGACCAATCAATCTGAAGCCATCTTAGCTTTCCCCAAGTGCTCCTCCTACCCGGATCAGCCAACGCCCACATACCTCAGGCTTAAACCAACTAGGGAACTTTCCAGTACTTTCCCAAACAAGGACCTACTGAGCCTTTCAGGTTCACAATCAATCAGATCCCTACTGGCTCACCTAGTCTCCCGACGCCTTCGCTTCAGTTTGGAAACGTCCAGATTACGCAGCCCCAGCGAGTAGGTGGGGGCTCCCTCAATATCAAACTGCACAACCGGGGTCCCCCCACCCCCCACCCCGTCCCTCCCTGCAAATTTGAGACGGCTCCAACTCAGTAATCTTTTTCCAAACTGGCCCATGAGGTCAGAGACAGTATCTCCATTGTAACGTGGCCGGGCGGTGTCAACACAAACGCCCCCACCCTCCCCTGGACGCGCGTAACCCGCTCCCCGCACCAGCCCCCTGCCCACAACTGCGCAGGCCCAGCAAGCCCCCACAATTAAAAGCCCAGCGCCGACCCTTCCTGTCAATTAGGCGCTGAAGCGCAGGCGGTCAGCATCGCCATGGAGACCAACACCCTTCCCACCGCCACTCCCCCTTCCTCTCAGGGTCCCTGTCCCCTCCAGTGAATCCCAGAAGACTCTGGAGAGTTCTGAGCAGGGGGCGGCACTCTGGCCTCTGATTGGTCCAAGGAAGGCTGGGGGGCAGGACGGGAGGCGAAAACCCTGGAATATTCCCGACCTGGCAGCCTCATCGAGCTCGGTGATTGGCTCAGAAGGGAAAAGGCGGGTCTCCGTGACGACT**TATAAA**AGCCCAGGGGCAAGCGGTCCGGATAACGGCTAGCCTGAGGAGCTGCTGCGACAGTCCACTACCTTTTTCGAGAGTGACTCCCGTTGTCCCAAGGCTTCCCAGAGCGAACCTGTGCGGCTGCAGGCACCGGCGCGTCGAGTTTCCGGCGTCCGGAAGGACCGAGCTCTTCTCGCGGATCCAGTGTTCCGTTTCCAGCCCCCAATCTCAGAGCGGAGCCGACAGAGAGCAGGGAACCGGC**ATG**

**HSPA6 Promoter (-1119🡪-114)**

CACCGGGCCTCTGGAGACGAGGCTCCTCGGGGATACAAACAGTGGGGAGAACATGAGGGACATCCCGACCGTACTCTGCGTCCTCCTTTCCCAGGTGTTGCGTTCTCTCTTGGGCTGAGTGGCGAGGTCTCTCCCGAGTCCCAGGGCCACAGTGCAATGTCACATCTCCTTTGTGGAAAGTGACTGGTAAAGGAGAGAGAACAAAACTGGAGGAATGTAAAGTCTTCAGCCACCTGGTTTAATTTATTCAAGAGTGATTAATCCTAGATGAGAAAAAGAATTGAAATGGATCGGAAAAAAATGAAAGTGCATTGGCCGGGAATCGAACCCGGGCCTCCCGCGTGGCAGGCGAGAATTCTACCACTGAACCACCAATGCTACTGTCAGCTAAAGACCTGCAGTATTGTCTCTTAAAGCTCACTATCTCTGGCCATTCACTAAGGAACCAGGCACCGTCTTAAATCGCGGTTTGGAAAATATTTTGTTCAAGATAAAACTGTTTTAAGATATACGTGTATATATCTTATATATCTGTATTCGCATGGTAACATATCTTCGGCCTTCCTGAGCCGCTGGGCTCTCAGCGGCCCTCCAAGGCAGCCCGCAGGCCCCTGTGTGCCTCAGGGATCCGACCTCCCACAGCCCCGGGGAGACCTTGCCTCTAAAGTTGCTGCTTTTGCAGCCTCTGCCACAACCGCGCGTCCTCAGAGCCAGCCCGGAGGAGCTAGAACCTTCCCCGCATTTCTTTCAGCAGCCTGAGTCAGAGGCGGGCTGGCCTGGCGTAGCCGCCCAGCCTCGCGGCTCATGCCCCGATCTGCCCGAACCTTCTCCCGGGGTCAGCGCCGCGCCGCGCCACCCGGCTGAGTCAGCCCGGGCGGGCGAGAGGCTCTCAACTGGGCGGGAAGGTGCGGGAAGGTGCGGAAAGGTTCGCGAAAGTTCGCGGCGGCGGGGGTCGGGTGAGGCGCAAAAGG**ATAAAAA**GCCGGTGGAAGCGGAGCTGAGCAGATCCGAGCCGGGCTGGCTGCAGAGAAACCGCAGGGAGAGCCTCACTGCTGAGCGCCCCTCGACGGCGGAGCGGCAGCAGCCTCCGTGGCCTCCAGCATCCGACAAGAAGCTTCAGCC**ATG**

**DHRS2 promoter sequence (Partial) (-3329 🡪 -2313)**

TAATGCCAAATCATTTCCCAAAGTGATTGTACTTACCCTCTACCCAGCAGTGAATGAGAGTTTCTGTTGATTTTGCTTTTTCAACTAGAAGTGAGAGGTGACAATATGCTAGCAGCCCTTGCTCACTCTTGGTGCCTCCTCGTCCTCCACATCCACTCTGGCTGCACTTGAGGAGCCCTTCAGCCCACCACTGCACTGTGGGGGCCCCTCTCTGGGGCTGGCCAAGGCCGAAGCCAGCTCCCTCTGCTCGCCAGGAGGTGTGGAGGGAGAGGCGCCGGCAGGAGCCCACACTGTACAGGGCACTCACCGGCCAGCAGGGGCTCCGTGGGCCGGCCGGTGCCAGCTGGGCCTGACTGGGGGATGAGCTCCCTCTGGGCTGCTGGAGTGCCTGGGCTAGGTGCCGCAAAGTCCTGCAGTGTGTGCCATTGAGAGGTGAAGCCGGCTGGGCTTCTGGGTCCGGTGGGGACCTGGAGAACTTTTCTGTCTAGCTAAAGGTTTGTAAATGCACCCATCAGCACTCTGTGTCTAGCTAAAGGTTTGTAAATGCACCAATCAGCAATCTGTGTCTAGCCAATCTGGTGGGGACTTGGAGAACTTTTGTGTCTAGCTAAAGGATTGTAAATGCACCAATCAGCACTCTGTGTCTAGCTAAAGGTTTGTAAATGCACCAATCAGCACTCTGTCAAAACGGACCAATCAGCTCTCTGTAAAACAGACCAATCAGCTCTCTGTAAAATGGACCAATCAGCTCTCCATAAAATGGACCAATCAGCAGGATGTGGGTGTGGCCAGTTAAGGGAATAAAAGCAGGCTGCCTGAGCCGGCAGCAGCAACCTGCTCTGGTTCCCTTCCACGCTGTGGAAGCTTTGTTCTTTTGGTCTTCATGATAAATCTTGCTGCTGCTCACTCGTTGGGTCCGTGCCACCTTTAAGAGCTGTAACACTCACCGCGAAGGTCTGCAACTTCACTCCTGGGGCCAGCAAGACCACGAATGCACCGAGAGGAATGAACAACTCTGGACACACCATCTTTAAGAACCGTAATACTCACCGCAAGGGTCTGCAACTTCATTCTTGAAGTCAGTGAGGCCAAGAACCCATCAATTCCGTACACATTTTGGTGACTTTGAAGAGACTGTCACCTATCACCAAGTGGTGAGACTATTGCCAAGCAGTGAGACTATTGCCAAGTGGTGAGACCATCACCAAGCGGTGAGACTATCACCTATCGCCAAGTGGTGAGTACCATCAGATCCCTTTCATTTGCTATTCTGTCCTATTTTTCCTTAGAATTCGGTGGCTAAATTCTGGGCACCTGTCGGCCAGTTAAAAGTGACTAGCGCAGCCGCTGGACTAAAAACGTGGGTGTCAGGCTTTCTGGGAAAGGGATCTCTAACAACCCCTGGCTCTGTGGAGTTGGGAATGTTGTTTTGCCTGGAACCAGCTTCCGCTTTTCCTGTACTTCTGGGCTGAGCTGAGGGTCAACAGAGAGGAAAGCCATTCAGCTCCGGAGTCCCCACAACAAGTTGGTTGACCCTGCGGCCATGAGCGGAACTCTCAAAGGCATGTTGCCCAAGTGAGACTCACCTATCTATCCTATCTATCCTGACCCTTGCTCGCTGGGTCCTAATGCCTGCCAGACAAACTTTCTCTCTCCTCTCTTCTCCTAGGCTAGTCCCACTTCTAAAAAACCACTCCCTGTCTCTGGTGCTTTTCTAATTTCTCTTATAAGAATGATTTCTAGTAAAAATTTCAGGACTCTGTTACCTTCTTTAGGCACCTGGGCTCACCAATCAGAAAGACATAATTTTTGCCCAAAGCCCAGTTGTAGGGGGAACTATCTGGAATTTTAGAATCCCTCCTCAGATAAGCAGGTCTAACAAAAGCTATTCCTGAAGCTAGGATAGGGGGAGCCTCAGAAATTGTATCCTTCCTATTCATATAAGTGAGGACAAAAGGTGTCACTTTTCCAACCCTGGAGACCCCTTCCCTCCCTCAGGTTACTCTTCTTCATTTTTGGGGCATAACATCTTTATAGGACACGGGTAAGTTCCCAATACTAACAGGAGAATGCTTAGGACTCTAACAGGTTTTCGAGAATGCGTCGGTAAGGGCCACTAAATCCGATTTTTCTCGGTCCTCCTCATGGTCTAGGAGGACAAGCAAGGGTGCAGCACTCTATGTCTAGCTAATCAGGTGGGGACTTGGAGAACTTTTGTGTCTAGCGAAAGGATTGTAAATGCACCAATCAGCACTCTGTGTCTAGCTAAAGGTTTGTAAACACAGCAATCAGCACTCTGTCAAAATGGACCAATCAGCTCTC**TATAAA**ACAGACCAATCAGCTCTCTGTAAAATGGACCAATCAGCAGGATGTGGACAGGGCCAGATAAGGGAATAAAAGCAGGCCACCCGAGCCAGCAGCAGCAACCTGCTTGAGTCCCCTTCCACACTGTGGAAGTTTTGTTCTTTCGCTCTTTGCAATAAATCTTGATTCACTGCTGCTCACTCTTTGGGTCCGCGCTGCCTTTAAGAGCTGTAACACTCACCACGAAGGTCTGCAACTTCACTCCTGAGGCCAGTGAGACCACAAACCCTCCAGAAGGAATGAACAACTCCAGACGCACTGCCTTTAAGAGCTGTAACACTCACTGCGAAGGTCTGCAGCTTCACTCCTGAAGCCAGCAAGACCACTAACCCACCAGGAGGAATGAACAACTCCGGACGGGAGGAATGAACAACTCCGGATGGGAGGAATGAACAACTCCGGACACACCATCTTTAAGAACTGTAACACTCATTGCGAGGGTCCGTGGCTTCATTCTTGAAGTCAGCGAGACCAAGAACCCACCAATTCCAGACACAGAAGCAGTTAATTAATAACTGGTATAATTATTTGTTGGTGCCTGGCATCTCATTTAACTGGGAAGCTGCATGGTTGGGGTCCAGGGCTGAGTCTGTCGTTTACTAAGGTGTCCCCGCCTCTACTGCCCAGTGCTTGACACATTGCCAGTGCTCAATGTTTGCTATGCAATGGATGGAAAAATCACCCTTGTCTAATGAATGTTGAGTCTCACATTTTAATTTGTGAATAATTTCCCCAGTTTTATAGCTTAGGAGTTTTCATGGATTGCTTTCCTGACCTGAGGTTCACATGTTTGAAATTTACCCTAACCAGCCTGACTCTCTGCCACTTTCTGTTGCTGGCCTTGTCTGTCTGGAGGAAGGAGGAGGGTAGATTACCTTCATGCTCACTGAGGCATCAGTGATAAGTGAAATTGATTCTTTCCCCCAGGCCTGATTCAGCAGGAAGCATCTCAGACACCAACCACT**ATG**

**FOXR1 promoter region (632bp)**

**HSF1 binding site (-857🡪 -226)**

GTCCCCCAGGCTGGAGTGCAGTGGCACCATCTCGGCTCACTGCAACCTCCACCTCCCGGGTTTAAGCGATTCTCCTGCCTCAGCCTCCCTAGTAGCTGGGATTACAGGCGTGAGCCACCACGCACTGCTAATTTTTTTGTATTTTTAGTAGAAACGGGGTCTCACCATCGGTCAGGCTGGTCTCAAACTTCTGACCTCAGGTGATCCGTCCACCTCGGCCTCCCAGAGTGTTGGGATTACATGCGTGAACCACCGCATGCGGCCAAAAGACAAAAAAAAATTTTTTTAATCAAATCACCATCCAATGTAATTCCATCTCCACCATCTACTACAGATGTGATCGTGGATAAACCATAAACCTTTCATAACTTCTAGAAAGTAACTACTAATACTATTATGGTAGAAAGGATCAATGTTAAAGCAAAAC**TATAAA**AATAAATAAATACGATCACTTGACCATGGCGCCTTTTCAATGAAATAAGGGGCAAGGGGCTTGGGCAAACGAGGATGCGTAAGATGATCTGGGGTCCCTTCCGGATCCCCAGGTCCTCGACCCCCGGCATGTAAGGGTAGAGTCAAGGAGGGTCTTCTTAAGCGTTCCTCGGCGCGGTCCTGAAGAGATTAAAGGCGTCAAATGGACCGCTCCACACCTGAGCTGCCGCCAAGCTAACTCAATCCGAGCCGGCGCATTTGAGAAGGCGCCTGTGAGGGTCGCTCCTCAGCCGCCGCGCTCCCACTCCGCGTCCCCACTCCGCGCCGCCGCGCCTCTGCCAGCCCCGAAGGTGGACGTGAAGCTCCAACACCTCGACTTCTGGGCCCGCCTCCATGGCCAGGTCCCGGGACTGCTGGACTGGGAC**ATG**
